# Electroencephalography-based biological and functional characteristics of spinal cord injury patients with neuropathic pain and numbness

**DOI:** 10.3389/fnins.2024.1356858

**Published:** 2024-05-01

**Authors:** Dezheng Wang, Xinting Zhang, Chen Xin, Chongfeng Wang, Shouwei Yue, Dongju Guo, Wei Wang, Yang Zhang, Fangzhou Xu

**Affiliations:** ^1^Rehabilitation and Physical Therapy Department, Shandong University of Traditional Chinese Medicine Affiliated Hospital, Jinan, China; ^2^Rehabilitation Center, Qilu Hospital of Shandong University, Jinan, Shandong, China; ^3^Department of Pediatrics, Qilu Hospital of Shandong University, Jinan, China; ^4^International School for Optoelectronic Engineering, Qilu University of Technology (Shandong Academy of Sciences), Jinan, China

**Keywords:** spinal cord injury, neuropathic pain, numbness, electroencephalogram, biological and functional characteristics, motor imagination

## Abstract

**Objectives:**

To identify potential treatment targets for spinal cord injury (SCI)-related neuropathic pain (NP) by analysing the differences in electroencephalogram (EEG) and brain network connections among SCI patients with NP or numbness.

**Participants and methods:**

The EEG signals during rest, as well as left- and right-hand and feet motor imagination (MI), were recorded. The power spectral density (PSD) of the θ (4–8 Hz), α (8–12 Hz), and β (13–30 Hz) bands was calculated by applying Continuous Wavelet Transform (CWT) and Modified S-transform (MST) to the data. We used 21 electrodes as network nodes and performed statistical measurements of the phase synchronisation between two brain regions using a phase-locking value, which captures nonlinear phase synchronisation.

**Results:**

The specificity of the MST algorithm was higher than that of the CWT. Widespread non-lateralised event-related synchronization was observed in both groups during the left- and right-hand MI. The PWP (patients with pain) group had lower θ and α bands PSD values in multiple channels of regions including the frontal, premotor, motor, and temporal regions compared with the PWN (patients with numbness) group (all *p* < 0.05), but higher β band PSD values in multiple channels of regions including the frontal, premotor, motor, and parietal region compared with the PWN group (all *p* < 0.05). During left-hand and feet MI, in the lower frequency bands (θ and α bands), the brain network connections of the PWP group were significantly weaker than the PWN group except for the frontal region. Conversely, in the higher frequency bands (β band), the brain network connections of the PWP group were significantly stronger in all regions than the PWN group.

**Conclusion:**

The differences in the power of EEG and network connectivity in the frontal, premotor, motor, and temporal regions are potential biological and functional characteristics that can be used to distinguish NP from numbness. The differences in brain network connections between the two groups suggest that the distinct mechanisms for pain and numbness.

## 1 Introduction

Neuropathic pain (NP) remains a common complication following spinal cord injury (SCI) and is considered the most painful and debilitating symptom of SCI (Burke et al., 2017). Up to 53% of patients with SCI develop NP (Burke et al., 2017). Furthermore, the current treatment outcomes of NP remain poor, which significantly affects the daily activities and rehabilitation efficacy for patients with SCI (Saurí et al., 2017; Vuckovic et al., 2018).

Neuroimaging studies of patients with SCI have revealed that Wallerian degeneration occurs in the ascending sensory fibre bundles due to the interruption of nerve afferents caused by injury, inducing both structural and functional changes in sensory relay nuclei located in the brainstem (Calderone et al., 2024). Furthermore, SCI patients with NP exhibit reduced metabolism in the medial prefrontal region and heightened functional magnetic resonance imaging (fMRI) power spectral density values in the primary motor cortex (M1), premotor cortex, thalamus, and periaqueductal grey during rest state, with these changes positively correlates with pain intensity (Nardone et al., 2018; Park et al., 2020). Another fMRI study have demonstrated increased connectivity in brain networks between the prefrontal cortex and regions involved in sensory integration and multimodal processing in SCI patients with NP (Huynh et al., 2021). Tanigor et al. found that compared with healthy volunteers, blood oxygen level dependent (BOLD) responses in somatosensory cortex (e.g., insular and tegmental) were reduced in patients classified as hypoesthesia and hyperalgesia and were more pronounced in hyperalgesia than in hypoesthesia (Tanigor et al., 2022). Previous studies have indicated that pain is often associated with an increase in θ-band power, considered the primary electroencephalogram (EEG) signature of pain (Vuckovic et al., 2018). An EEG analysis of SCI patients with NP has also revealed heightened α band activity in the frontal region (Vuckovic et al., 2018). Hasan et al. proposed that pain induces alterations in the functional connections of the frontal–parietal network in the θ band, whereas changes in the functional connections of the sensory–motor network in the β band may be attributed to injury (Hasan et al., 2021). However, previous EEG studies have illustrated that decreased α band EEG power and the shift of major α frequencies to lower frequencies may be linked to NP (Vuckovic et al., 2018).

These findings underscore that the altered EEG activity and brain network function in SCI patients with NP, however, the available perspectives remain inconsistent. A growing body of evidence has suggested that pain does not originate from the activation of specific neurons or brain regions but rather is arises from interactions between a group of interconnected neurons (Mouraux and Iannetti, 2018). Central sensitisation occurs in the spinal cord of patients with various chronic pain (Arendt-Nielsen et al., 2018). However, it remains nebulous as to how the central nervous system changes and causes persistent pain in SCI patients when the peripheral input is diminished or absent. The most common characteristics of SCI include pricking pain, followed by pain and numbness (Kim et al., 2020). Numbness is associated with sensory deficits, whereas NP is characterized by hyperalgesia or allodynia. Therefore, it is feasible to determine the potential targets of SCI-related NP by determining whether pain or numbness following SCI results from different brain region activation or network connection changes. But there are still no studies on the EEG signal Characteristics about neuropathic pain and numbness after SCI.

The method of EEG analysis directly impacts the sensitivity and accuracy of the detection results; consequently, in-depth analyses of EEG have been extensively conducted to improve this technique. Multiparadigm methods, including those based on wavelets, nonlinear dynamics, and chaos theory, offer effective approaches for the automatic EEG signals diagnosis, they often require substantial data acquisition, presenting inherent limitations. And it has been proved that nonlinear dynamics and chaos are not suitable for research involving motion imagination of disabled people, because the participants have different degrees of impairment, and the use of nonlinear dynamics may affect the accuracy of the results (Calderone et al., 2024). The modified S-transform (MST) is an ideal method for EEG signals analysis and was therefore adopted in the present study. This method can dynamically adjust the window width and achieve superior energy concentration in the temporal–frequency domain (Xu et al., 2020, 2022). The MST algorithm exhibits enhanced recognition effects compared with other algorithms (Xu et al., 2020, 2022).

The primary objective of this study is to identify new potential targets for evaluation and intervention by examining and contrasting alterations in EEG signals and brain network connections during rest and task-performing states between SCI patients with numbness and those with NP.

## 2 Materials and methods

### 2.1 General information

Thirty-six patients diagnosed with SCI and admitted to Qilu Hospital of Shandong University from June 2020 to October 2023 were enrolled in the study. All patients had passed the spinal shock period and were categorized into two groups based on their predominant symptomatology: those with NP (PWP group, *n* = 18) and those with numbness (PWN group, *n* = 18). It is important to note that all patients had NP/numbness and motor dysfunction below the injury level.

The inclusion criteria for this study include the following: meeting the diagnostic criteria for SCI, having normal vision or normal vision after correction, possessing the ability to comprehend the tasks assigned, absence of neurological diseases or injuries besides SCI, and experiencing NP or numbness below the level of injury l persisting for a minimum of 4 weeks. The pain score of the PWP group was set to ≥5 on the Leeds Assessment of Neuropathic Symptoms and Signs scale (LANSS). The completeness of injury was assessed using the American Spinal Cord Injury Association (ASIA) Impairment Scale, categorized as A (complete injury), B (sensory incomplete), C (motor incomplete with more than half of key muscle functions below the neurological level of injury have a muscle grade < 3); and D (motor incomplete with at least half of key muscle functions below the neurological level of injury having a muscle grade ≥ 3) (Kirshblum et al., 2021). Since there is no established relationship between patient sex, age, degree of injury, completeness of the injury, and the incidence of NP and numbness (Vuckovic et al., 2018), participants of all sexes, with paraplegia and panplegia, and with complete or incomplete injuries, were included in the study. The mean age of the PWP and PWN groups is 48.06 ± 11.54 years and 52.28 ± 6.96 years, respectively, with no significant differences between the two groups (*p* > 0.05). Detailed information regarding the patient groups is listed presented Table 1. To avoid the effect of the drugs and rehabilitation on the EEG records of patients with pain and numbness, all patients had completed the EEG records before therapy was initiated. Following EEG recording, all patients received routine treatment to improve their symptoms. To exclude the effect of handedness on EEG signals, all the patients were right-handed.

**Table 1:** Demographic characteristics of the participants in the two groups.

Group	NO.	Sex	Age	Injury level	Completeness of injury	LANSS score	Month after injury	Group	NO.	Sex	Age	Injury level	Completeness of injury	Month after injury
				left/right								left/right		
PWP	1	M	26	T10/T10	D	5	2	PWN	1	M	49	C4/C4	D	1
	2	M	32	T12/T12	C	19	19		2	M	60	C3/C3	D	6
	3	M	43	T9/T9	A	5	2		3	M	50	C4/C5	D	24
	4	M	48	L5/L5	D	5	3		4	M	41	C4/C4	D	24
	5	M	59	T11/T8	D	5	3		5	M	49	C3/C5	D	3
	6	M	34	T12/L2	C	10	3		6	M	51	C3/C3	D	5
	7	F	61	T5/T5	D	8	2		7	M	48	C5/C5	D	12
	8	F	66	T7/T7	D	11	10		8	M	52	C5/C5	D	12
	9	F	42	T4/T4	C	16	7		9	M	56	C2/C3	D	6
	10	F	37	T9/T8	A	5	1		10	M	55	C3/C3	D	2
	11	M	59	T11/T11	C	10	3		11	M	63	C5/C5	D	12
	12	M	53	T10/T10	A	7	2		12	M	40	C4/C4	D	24
	13	F	39	T5/T5	D	8	1		13	M	42	C4/C4	D	3
	14	M	47	T11/T11	D	5	2		14	M	56	C4/C4	D	1
	15	F	51	C6/C6	D	7	4		15	M	55	C5/C5	D	1
	16	M	58	T4/T4	D	5	6		16	M	56	C3/C3	D	3
	17	F	48	L1/T10	C	7	9		17	M	65	C5/C5	D	20
	18	F	62	T8/T8	D	5	24		18	M	53	C3/C3	D	1

The participants’ sex is shown in Table 1; F, female. M, male; The completeness of injury was determined using the American Spinal Cord Injury Association (ASIA) Impairment Scale; A, complete injury; B, sensory incomplete; C, motor incomplete, more than half of key muscle functions below the neurological level of injury have a muscle grade less than 3; D, motor incomplete, at least half of key muscle functions below the neurological level of injury have a muscle grade ≥ 3; PWP, SCI patients with neuropathic pain; PWN, spinal cord injury patients with numbness.

The exclusion criteria for all participants included chronic or acute muscle or visceral pain, LANSS score of <4 in the PWP group, the presence of other major neurological disorders (i.e., stroke, traumatic brain injury, or epilepsy), a history of mental illness, and cognitive dysfunction. Prior to participation, all participants provided signed informed consent forms. This study was conducted with the approval of the Ethics Committee of Shandong University Qilu Hospital (KYLL-2020 (KS)-475).

### 2.2 Methods

#### 2.2.1 EEG acquisition instrument

The EEG recordings were conducted using the 64-256 NeuroScan EEG system (Australia) during the rest and task-performing states. The EEG electrodes were placed according to the standard 10-10 system with a connecting-ear reference and the ground electrode placed at the AFz location. Additionally, eye electrical signals were recorded from four channels positioned around both eyes. The EEG data were sampled across all channels at a frequency of 1,000 Hz, ensuring high temporal resolution, and the impedance of each electrode was maintained below 10 kΩ to optimize signal quality. The EEG data were sampled across all channels at a frequency of 1,000 Hz, ensuring high temporal resolution, and the impedance of each electrode was maintained below 10 kΩ to optimize signal quality.

#### 2.2.2 EEG information collection

##### 2.2.2.1 Resting-state electroencephalogram

During the resting state, participants were instructed to sit at a distance of approximately 1 m away from the computer screen. They underwent two-minute spontaneous electrical brain activity recording sessions, alternating between eyes open (EO) and eyes closed (EC) conditions, each repeated twice. In the resting state with EO, participants were instructed to maintain stillness and focus their gaze on a small crosshair presented in the centre of the computer screen, whereas in the resting state with EC, participants were instructed to be relaxed.

##### 2.2.2.2 Task-state electroencephalogram

**Figure 1:**
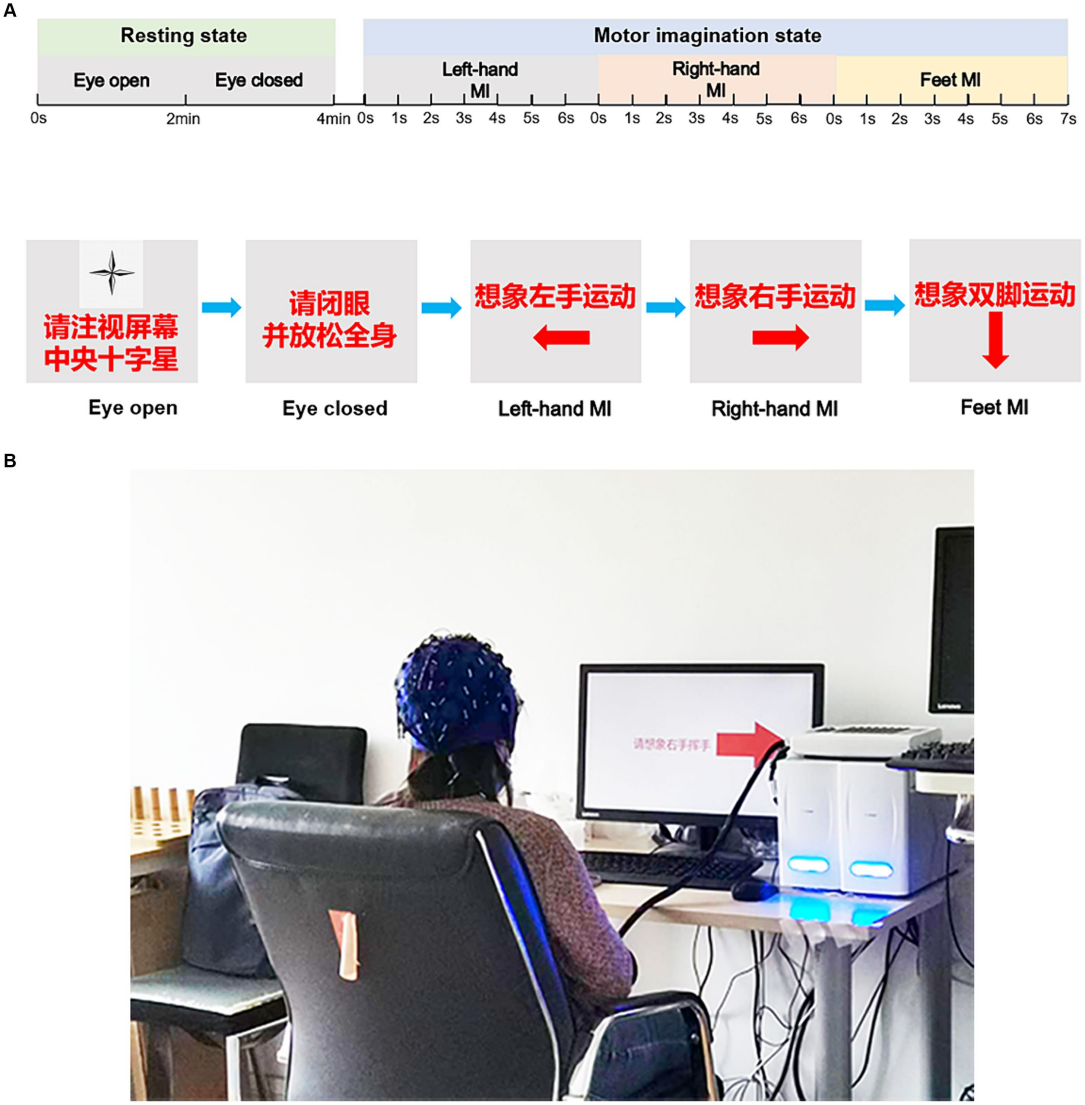
The experimental diagram. Experimental procedures for resting-state and task-state EEG recordings **(A)**. **(B)** Participants of the EEG recording. The participant provided written informed consent for publication of his image. EEG, electroencephalogram.

During motor imagination (MI) tasks, an arrow pointing right, left, or down randomly appeared in the centre of the screen during the initial 3 s of each trial. The participants were instructed to imagine performing specific motor actions based on the direction of the arrow: right-hand movement (text prompt and symbol →), left-hand movement (text prompt and symbol ←), or tapping with both feet (text prompt and symbol ↓). The cues appeared randomly across trials. A total of 120 trials (40 trials for each MI) were conducted in four experimental sub-phases within the same experimental session, with a 90 s break between each sub-phase. Each sub-phase included 30 trials and lasted approximately 5 min. The experimental procedure is illustrated in Figure 1 for clarity.

#### 2.2.3 Data processing

##### 2.2.3.1 Data pre-processing

EEG data were imported to EEGLAB v2019.0 (University of California, United States) in MATLAB (R2020a, Mathworks) to conduct channel positioning and re-reference using M1 and M2 as reference electrodes. The unusable electrode recordings and power frequency interference were removed. To isolate specific frequency bands, the Butterworth filter was used to select the θ (4–8 Hz), α (8–12 Hz), and β (13–30 Hz) bands. An independent component analysis algorithm was utilized to decompose the data into 60 independent temporal components across all frequency bands. EEG signals were examined, and those with electrical ocular artefacts were identified and removed. Finally, the obtained data were downsampled to 100 Hz for further analysis.

##### 2.2.3.2 Feature extraction

The Continuous Wavelet Transform (CWT) has many advantages such as multi-scale analysis, adaptability and high computational efficiency, while the MST has the advantages of high resolution, strong anti-interference and strong self-adaptability. These advantages make them widely used in different application fields, and play an important role in signal processing, image processing, audio processing and other fields. And in order to compare the advantages of MST compared with CWT, the CWT and MST were applied to the data using MATLAB to calculate the power spectra density (PSD). And MATLAB was used to normalise the data after feature extraction. The normalized data were averaged for statistical analysis. The equations for CWT and MST are provided in the Supplementary material.

##### 2.2.3.3 Brain network

The phase-locking value (PLV) was used for assessing phase synchronization between two brain regions. PLV can capture nonlinear phase synchronization (Chiarion et al., 2023). Suppose the instantaneous phase of the two signals *g*(*t*) and *p*(*t*) as *λg*(*t*), *λp*(*t*). The equation for PLV is provided in the Supplementary material. PLV has been extensively utilized to calculate the connection weights of each EEG segment in previous studies (Li et al., 2019).

### 2.3 Statistical analysis

The statistical analysis was performed using SPSS 25.0 software, while the power calculations were conducted with Pass 11.0 software. The measured data obeyed a normal distribution and are presented as the mean ± standard deviation. An independent sample *t*-test was used to employed to assess differences between the PWP and PWN groups, with the significance level set at *α* = 0.05. *Post hoc* power was calculated at 91.3% based on the PSD value. The mean (standard deviation) for PWP group was 9.8 (4.0), *n* = 18, while for the PWN group, it was 5.2 (4.3), *n* = 18. The *α* error level was set at 0.05 for bidirectional judgement.

## 3 Results

### 3.1 MI-associated biological characteristics

#### 3.1.1 Characteristic information extracted using CWT

**Figure 2:**
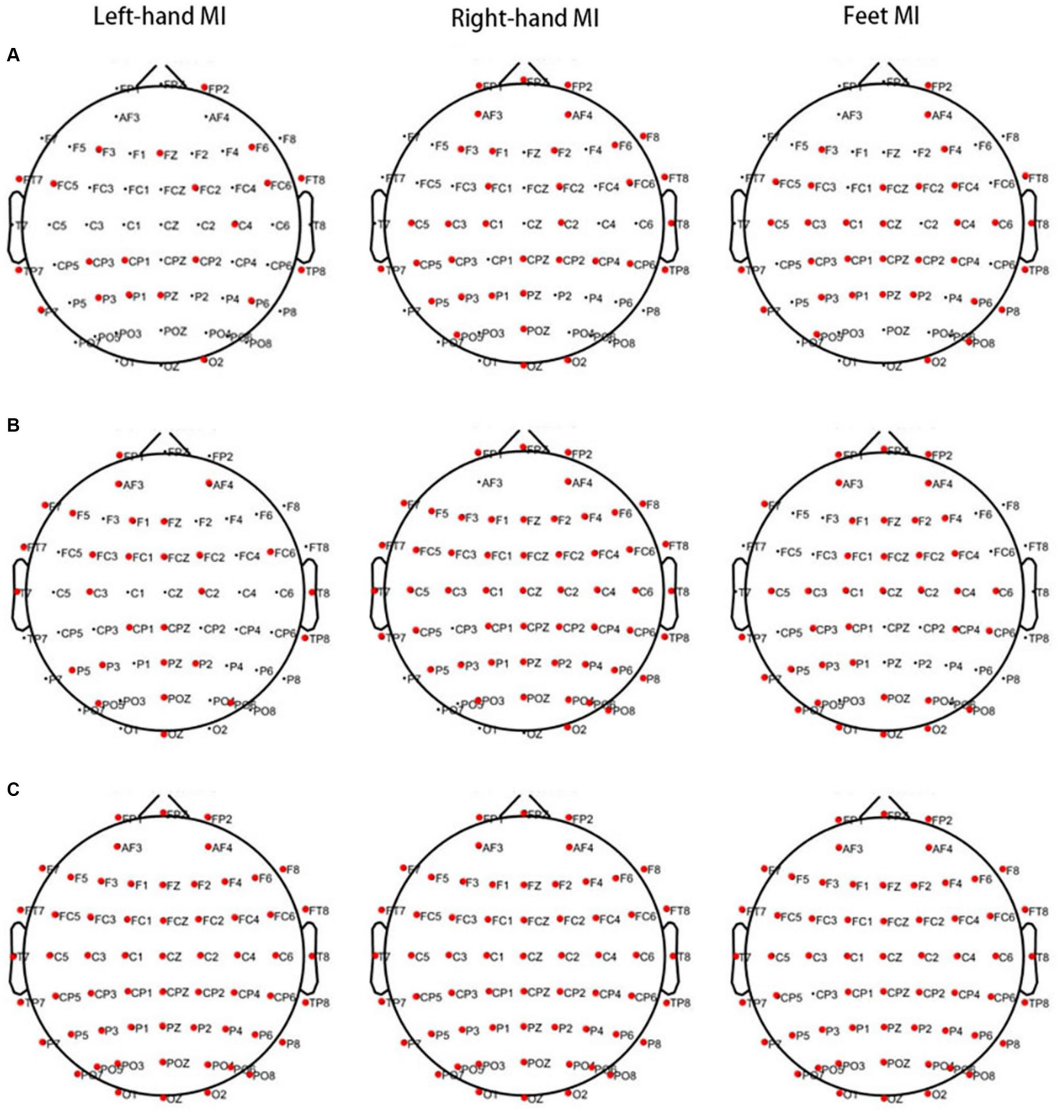
Areas showing a significant difference between MI-induced PSD values in the two groups analysed by CWT. The bold red dots represent channels with significant differences. When left- and right-hand, and feet MI is performed, the PSD values of the PWP group are significantly lower than those of the PWN group on multiple channels in the θ (**A**, all *p* < 0.05) and β bands (**C**, all *p* < 0.05) and is significantly higher than those of the PWN group in the α band (**B**, all *p* < 0.05). MI, motor imagination; PSD, power spectral density; CWT, continuous wavelet transform; PWP, spinal cord injury patients with pain; PWN, spinal cord injury patients with numbness.

Significant differences were observed in PSD values of 21 channels in the θ band, 28 channels in the α band, and all 60 channels in the β band between the PWP and PWN groups when left-hand MI was performed. When right-hand MI was performed, 35 channels in the θ band, 53 channels in the α band, and all 60 channels in the β band differed between the PWP and PWN groups. Finally, 34 channels in the θ band, 38 channels in the α band, and 59 channels, except for CP3, in the β band differed between the PWP and PWN groups when both-feet MI was performed (Figure 2).

#### 3.1.2 Characteristics information extracted by MST

**Figure 3:**
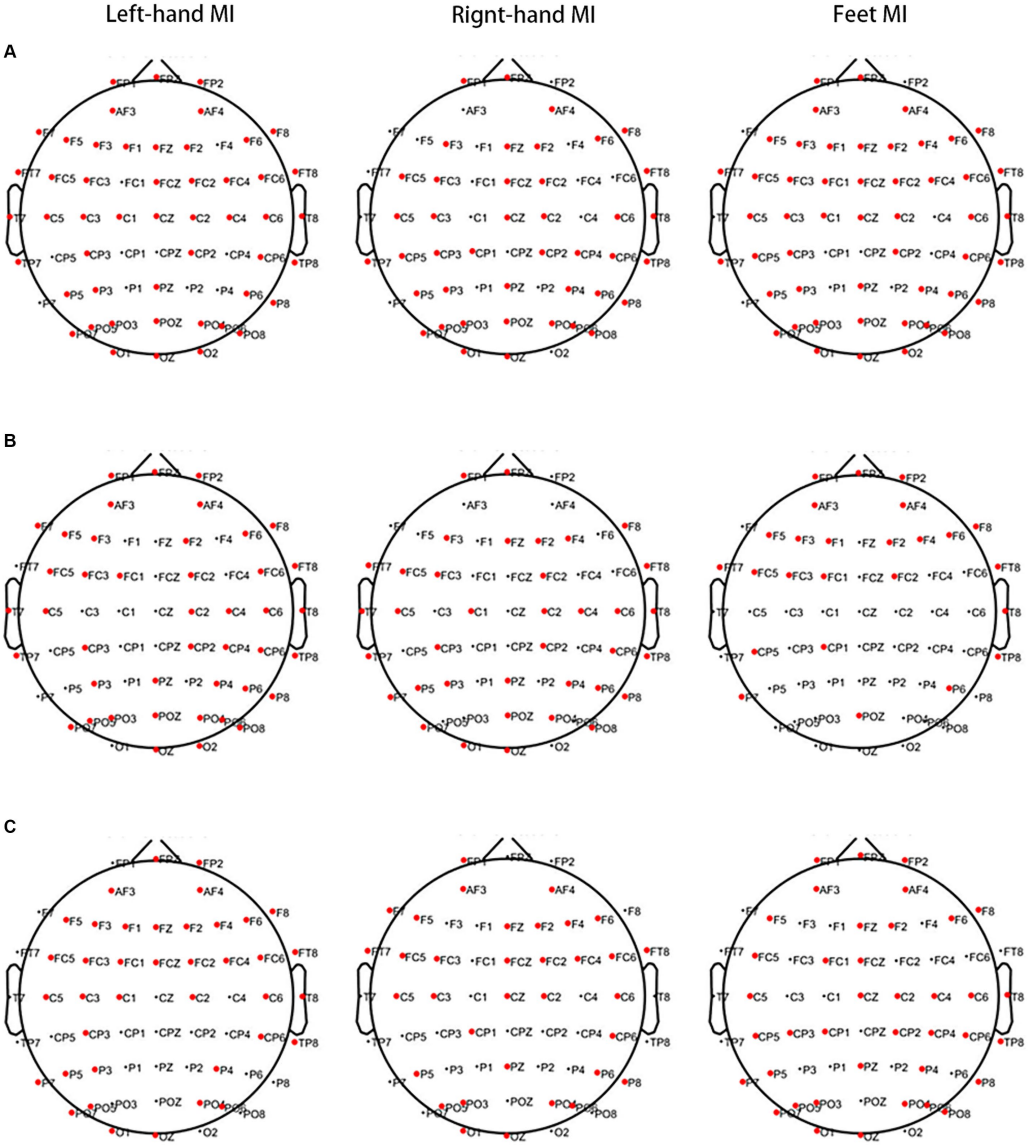
Areas showing a significant difference between MI-induced PSD values in the two groups analysed by MST. The bold red dots represent channels with significant differences. When left- and right-hand, and feet MI is performed, the PSD values of the PWP group are significantly lower than that of the PWN group on multiple channels in the θ (**A**, all *p* < 0.05) and α band (**B**, all *p* < 0.05) and are significantly higher than those of the PWN group in the β band (**C**, all *p* < 0.05). MI, motor imagination; PSD, power spectral density; MST, modified S-transform; PWP, spinal cord injury patients with pain; PWN, spinal cord injury patients with numbness.

When left-hand MI was performed, lower PSD values in 50 channels of the frontal, premotor, motor, temporal, parietal, and occipital regions in the θ band and 43 channels of the frontal, premotor, motor, temporal, parietal, and occipital regions in the α band were observed in the PWP than PWN groups. Higher PSD values were observed in 39 channels of the frontal, premotor, motor, temporal, parietal, and occipital regions in the β band of the PWP than in the PWN groups. When right-hand MI was performed, lower PSD values in 42 channels of the frontal, premotor, motor, temporal, parietal, and occipital regions in the θ band and 37 channels of the frontal, premotor, motor, temporal, parietal, and occipital regions in the α band were observed in the PWP than PWN groups. Furthermore, higher PSD values in 34 channels of the frontal, premotor, motor, parietal, and occipital regions in the β band were observed in the PWP group than in the PWN group. When feet MI was performed, lower PSD values in 50 channels of the frontal, premotor, motor, temporal, parietal, and occipital regions in the θ band and 25 channels of the frontal, premotor, temporal, parietal, and occipital regions in the α band were observed in the PWP than PWN groups. Higher PSD values were observed in 39 channels of the frontal, premotor, motor, temporal, parietal, and occipital regions in the β band in the PWP than in the PWN groups (Figure 3).

### 3.2 Differences in spontaneous EEG activities between the two groups

**Figure 4:**
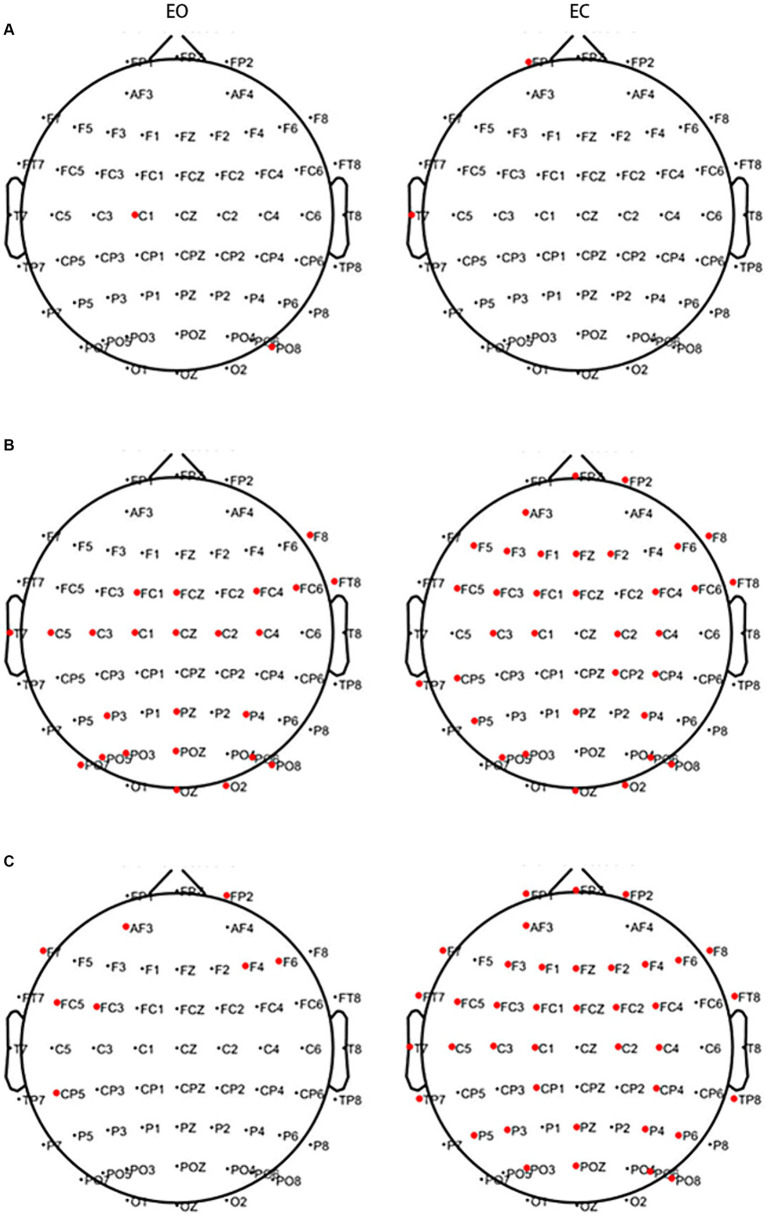
Areas showing a significant difference between spontaneous EEG activity in the two groups. The bold red dots represent channels with significant differences. In the EC and EO states, in both the θ **(A)** and α bands **(B)**, the PSD values of the PWP group on multiple channels are significantly lower than those of the PWN group (all *p* < 0.05) and are significantly higher than those of the PWN group in the β band (**C**, all *p* < 0.05). EC, eyes closed; EO, eyes open, PSD, power spectral density; PWP, spinal cord injury patients with pain; PWN, spinal cord injury patients with numbness.

The above results suggest that CWT is not specific; therefore, the MST algorithm was used for analysis. In the EO state, lower PSD values in two channels of the motor and parietal regions in the θ band and the 25 channels of the frontal, premotor, motor, temporal, and occipital regions in the α band were observed in the PWP than PWN groups. Higher PSD values in the eight channels of the frontal, premotor, and parietal regions in the β band were observed in the PWP group than in the PWN group. Conversely, in the EC state, lower PSD values in two channels of the frontal and temporal regions in the θ band and 34 channels of the frontal, premotor, motor, parietal, and occipital regions in the α band were observed in the PWP than PWN groups. Higher PSD values in 39 channels of the frontal, premotor, motor, temporal, and parietal regions in the β band were observed in the PWP group than in the PWN group (Figure 4).

### 3.3 Event-related synchronization and desynchronization

**Figure 5:**
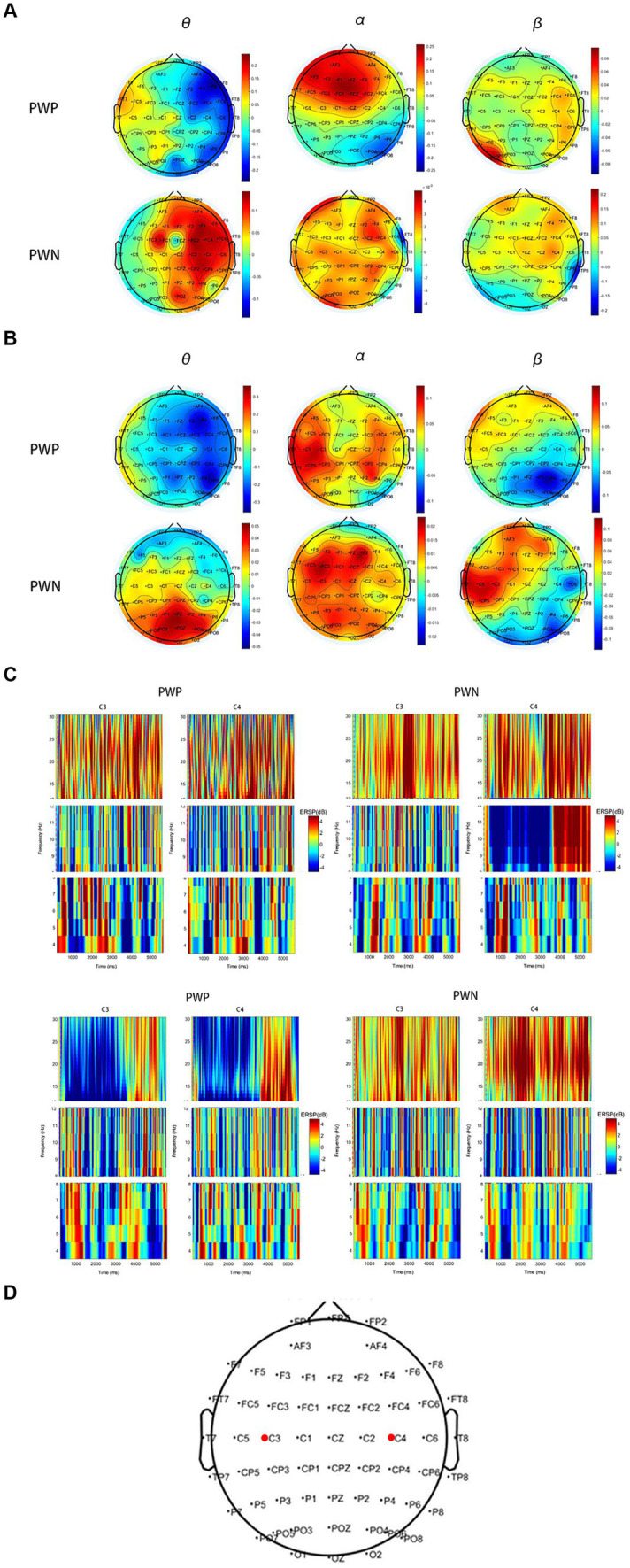
ERS and ERD during performing left- and right-hand MI. **(A,B)** are scalp maps based on ERD/ERS for the PWP and PWN groups. Each dot represents an electrode. **(C,D)** are the time-frequency graphs of cue-based MI. Cortical activity averages in the θ (4–8 Hz), α (8–12 Hz), and β (13–30 Hz) frequency bands during MI of the left **(A,C)** and right hand **(B,D)**. **(E)** shows the locations of C3 and C4. Positive values (red) indicate ERS and negative values (blue) indicate ERD. The bold red dots represent the location of C3 and C4. ERS, event-related synchronization; ERD, event-related de-synchronization; PWP, spinal cord injury patients with pain; PWN, spinal cord injury patients with numbness, MI, motor imagination.

During left-hand MI, the PWP group showed ERS in the ipsilateral brain region and ERD in the ipsilateral brain region in the θ and α bands, whereas the PWN group showed significant ERS in the θ band and non-lateralised ERS in the α band. Conversely, during right-hand MI, the PWP group showed non-lateralised ERD in the θ band and non-lateralised ERS in the α band, whereas the PWN group showed significant non-lateralisation ERS in the θ and α bands. Finally, during left- and right-hand MI, both groups showed more significant ipsilateral ERS compared with the contralateral brain region and more significant contralateral compared with the ipsilateral brain region ERD in the β band (Figure 5).

### 3.4 Comparison of functional connectivity between the two groups

**Figure 6:**
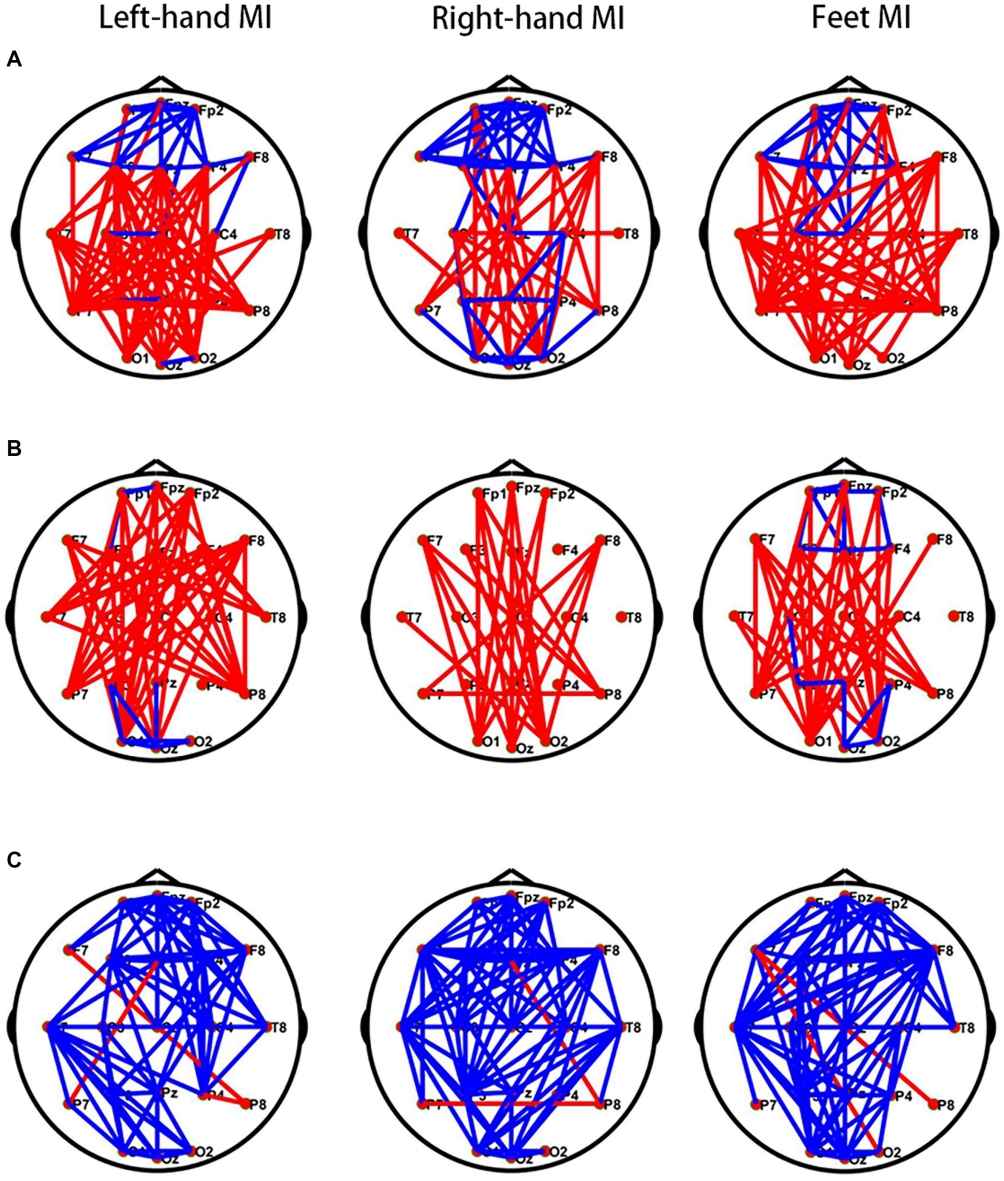
Comparison of brain networks between the PWP and PWN groups. Comparison of **(A)** the θ, **(B)** α, and **(C)** β band brain network connections between the PWP and PWN groups; the blue line indicates increased connection, and the red line indicates decreased connection. PWP, spinal cord injury patients with pain; PWN, spinal cord injury patients with numbness.

During left- and right-hand and feet MI, the frontal region in the θ band of the PWP group showed significantly stronger brain network connections than those of the PWN group; however, the other regions in the θ band of the PWP group showed weaker brain network connections. During left-hand and feet MI, in the α band, the frontal region of the PWP group showed stronger brain network connections than those of the PWN group; however, the other regions of the PWP group showed weaker brain network connections. During right-hand MI, in the α band, all brain regions of the PWP group showed weaker brain network connections than those of the PWN group. During left- and right-hand and feet MI, all brain network connections in the β band of the PWP group were significantly stronger than those of the PWN group (Figure 6).

## 4 Discussion

According to results, it can be found that there are more differential channels after CWT processing than MST, which proves that CWT has high sensitivity but low specificity. However, only some channels of MST have differences, which proves that MST has strong specificity. Therefore, in this study, the MST algorithm was used to extract information on EEG characteristics information owing to its higher specificity compared with CWT. Significant differences were observed in the EEG activities in the frontal, premotor, motor, and temporal regions and the functional connectivity of the brain network between the SCI patients with NP or numbness during the MI and resting states.

Cortical reorganisation of the M1 and primary somatosensory cortex following SCI has been shown to be associated with motor and sensory impairment (Park et al., 2020). To predict the occurrence and prognosis of NP following SCI, previous studies have detected differences in EEG signals between SCI patients with NP and those without NP or healthy controls. However, there are only a few studies on changes in brain function in patients with numbness following SCI. In a previous study, injurious stimuli were administered to patients with hypaesthesia, patients with hyperalgesia, and healthy controls and fMRI was used to assess the activities of various brain regions (Tanigor et al., 2022). The findings revealed that the decreased activity during stimulation in patients with hypoesthesia compared with healthy volunteers was localised in the postcentral gyrus, whereas patients with hyperalgesia exhibited decreased activity in the precentral and postcentral gyri, insular cortex, and operculum. These central changes were suggested to be associated with deafferentation (Tanigor et al. 2022). The findings also suggested that the absence of afferents might have led to altered neuroplasticity in these patient groups, resulting in functional and structural changes (Tanigor et al. 2022). Additionally, Akimoto et al. demonstrated that the increased input drive from two abnormal sensations, pain and numbness, into the brain is followed by a higher oscillation amplitude of neuronal activity (Akimoto et al., 2024). However, the authors did not determine whether the afference of the two abnormal sensations had different effects on the brain.

In this study, analysis of the EEG signals from SCI patients experiencing either numbness or NP revealed that the PSD values of the PWP group were significantly lower than those of the PWN group in the θ and α bands during the resting and MI states. The β-band PSD values of the PWP group were significantly higher than those of the PWN group. Similar to the findings of Takenaka et al. (2020), compared with SCI patients with NP, the cortical areas with significant differences in EEG signals in patients with numbness were primarily the frontal, premotor, and motor regions during MI, suggesting that the afference of two abnormal sensations, pain and numbness, differently affects brain activation. Furthermore, the results also revealed that when MI was performed, the regions of the cerebral cortex with distinct electrical activity between patients with numbness and those with pain also included the temporal region, which is implicated in functions such as emotion and memory. This indicating that numbness and pain following SCI may have disparate effects on patients’ emotions states. Moreover, the observed reduction in θ and α bands and elevation in the β band of the EEG power in the frontal, premotor, motor, and temporal regions of NP patients compared with those with numbness can be used as sensitive biological characteristics for distinguishing numbness from pain. The frontal, premotor, motor, and temporal regions represent potential therapeutic targets for non-invasive neuro-regulation of NP and numbness post-SCI.

In the θ and α bands, patients in both groups exhibited a widespread non-lateralised ERS phenomenon, which might be associated with abnormal activation or inhibition of brain regions due to nerve injury rather than pain or numbness (Osuagwu et al., 2016). However, both groups showed non-significant ERS and ERD in the β band. The precise underlying mechanism behind this phenomenon warrants further investigation and remains to be elucidated.

To delve deeper into the central distinctions between numbness and pain, the functional connectivity of brain networks was examined. During left-hand and both-feet MI, in the lower frequency bands, namely the θ and α bands, the functional connections of the frontal region were significantly enhanced in the PWP group compared with the PWN group, whereas the functional connections of other brain regions were significantly weakened. This suggests that pain enhances the functional connections of the frontal region in the lower frequency bands while concurrently inhibiting the functional connections between other brain regions in the same frequency band. NP may activate the salience network (SN) located in the frontal region (Marek and Dosenbach, 2019; Hasan et al., 2021). During stimulation with visual or auditory stimuli, the SN activates the executive network, suppresses the default mode network, and can be strongly activated in the rest-to-task switch state (Hasan et al., 2021; Jiang et al., 2023). Previous studies have shown that the SN is involved in both task execution and pain processing (Hasan et al., 2021), and higher pain intensity can enhance the functional connectivity of the SN (van Ettinger-Veenstra et al., 2019), consistent with our results. Additionally, in the higher-frequency band, such as the β band, the connections between all brain regions in the PWP group were significantly intensified compared to the PWN group, suggesting that the network connections between brain regions in the high-frequency band are activated by pain or inhibited by numbness. These results illustrates the significant differences in the effects of pain and numbness on the functional connections between various brain regions, indicating potential differences in the central mechanisms of numbness and pain.

Investigating the differences in the EEG signals between the SCI patients experiencing NP or numbness not only sheds light on the origins of these two abnormal sensations but also provide a potential objective tool for assessing NP. Currently, available questionnaires for evaluating patients’ discomfort are subjective due to various factors. Researchers are committed to developing neurotechnologies to improve the diagnosis and treatment of NP (Birch et al., 2022). The observed reductions in θ and α band, coupled with increases in β band of the EEG power in the frontal, premotor, motor, and temporal regions, along with the weaker brain network connections in θ and α bands and stronger brain network connections in the β band of patients with NP compared to patients with numbness can objectively distinguish NP from numbness. These findings hold promise as potential biological and functional characteristics for use in clinical settings.

The study has some limitations. The number of participants in each group is relatively small, and there is no quantitative analysis of the EEG difference between neuropathic pain and numbness after spinal cord injury. Although we have avoided variables that could affect brain function as much as possible in the study, there are still some uncontrollable factors, such as the course of disease and dominant hand. In the future, we will conduct larger-scale clinical studies to validate our experimental conclusions. And we also will combine the research conclusions with treatment to guide clinical treatment.

## 5 Conclusion

Our study demonstrated that the reduced EEG activities of θ and α bands in the frontal, premotor, motor temporal, parietal, and occipital regions and the enhanced EEG activities of the β band in the same brain regions of patients with NP during both MI and resting states, compared with numbness patients, can be used in clinical settings as potential biological and functional characteristics to distinguish NP from numbness. The weaker brain network connections of the θ and α bands and the stronger brain network connections of the β band in the frontal, premotor, motor, and temporal regions were observed in patients with NP compared to those with numbness. These differences in brain network connections were identified between two groups of patients, suggesting that the distinct mechanisms of pain and numbness. These findings will guide the differential diagnosis of neuropathic pain and numbness after spinal cord injury and further non-invasive neuroregulatory therapy and drug therapy.

## Data availability statement

The raw data supporting the conclusions of this article will be made available by the authors, without undue reservation.

## Ethics statement

The studies involving humans were approved by the Ethics Committee of Shandong University Qilu Hospital, KYLL-2020 (KS)-475. The studies were conducted in accordance with the local legislation and institutional requirements. The participants provided their written informed consent to participate in this study. Written informed consent was obtained from the individual(s) for the publication of any potentially identifiable images or data included in this article.

## Author contributions

DW: Writing – original draft. XZ: Writing – original draft. CX: Writing – review & editing. CW: Writing – review & editing. SY: Writing – review & editing. DG: Writing – review & editing. WW: Writing – review & editing. YZ: Writing – review & editing. FX: Writing – review & editing.
