# Supplementary material for: Electroencephalography-based biological and functional characteristics of spinal cord injury patients with neuropathic pain and numbness
Source: Front Neurosci. 2024 May 1;18:1356858. doi: 10.3389/fnins.2024.1356858 (PMC11094546; doi:10.3389/fnins.2024.1356858)
Supplement: Supplementary file 1 [file Data_Sheet_1.docx]

Supplementary Material

Electroencephalographic-Biomarkers of Neuropathic Pain and Numbness in Patients with Spinal Cord Injury

Dezheng Wang^1^, Xinting Zhang^2^, Chen Xin^1^, Chongfeng Wang^3^, Shouwei Yue^1^, Dongju Guo^1^, Wei Wang^1^, Fangzhou Xu^3^*, Yang Zhang^1^*

^1^ Rehabilitation Center, Qilu Hospital of Shandong University, Jinan, Shandong, China

^2^ Department of Pediatrics, Qilu Hospital of Shandong University, Jinan, Shandong, China

^3^ International School for Optoelectronic Engineering, Qilu University of Technology (Shandong Academy of Sciences), Jinan, China

Dezheng Wang and Xinting Zhang contributed equally.

*** Correspondence:**Fangzhou Xu and Yang Zhang, email: [xfz@qlu.edu.cn](mailto:xfz@qlu.edu.cn) and zhangyang982003@163.com

# Supplementary Figures and Tables

## Supplementary Figures

Supplementary Fig.1 The inclusion and exclusion flow chart of participant

## Supplementary Tables

72 patients with SCI met the inclusion criteria

51 patients were selected for the study

21 patients met the exclusion criteria:

13 patients with history of mental illness;

8 patients with cognitive dysfunction.

43 patients participated in the study

18 patients with NP and 18 patients with numbness were included in this study

8 patients refused to participate.

7 patients with both NP and numbness were excluded after assessing.

### The P and T values of characteristic information extracted by MST

**Supplementary table1. P and T value of θ band**

|  | Left-hand MI | | Right-hand MI | | Feet MI | | EO | | EC | |
| --- | --- | --- | --- | --- | --- | --- | --- | --- | --- | --- |
|  | P value | T value | P value | T value | P value | T value | P value | T value | P value | T value |
| FP1 | 0.000 | -6.084 | 0.000 | -4.439 | 0.000 | -7.937 |  |  | 0.014 | -2.589 |
| FPZ | 0.000 | -5.898 | 0.000 | -4.734 | 0.000 | -8.245 |  |  |  |  |
| FP2 | 0.005 | -2.843 |  |  |  |  |  |  |  |  |
| AF3 | 0.000 | -6.165 |  |  | 0.000 | -4.905 |  |  |  |  |
| AF4 | 0.000 | -8.013 | 0.005 | -2.816 | 0.000 | -3.927 |  |  |  |  |
| F7 | 0.000 | -4.505 |  |  |  |  |  |  |  |  |
| F5 | 0.000 | -6.171 |  |  | 0.000 | -4.106 |  |  |  |  |
| F3 | 0.000 | -9.294 | 0.000 | -5.137 | 0.000 | -5.915 |  |  |  |  |
| F1 | 0.024 | -2.261 |  |  | 0.001 | -3.267 |  |  |  |  |
| FZ | 0.000 | -6.810 | 0.000 | -4.435 | 0.000 | -7.044 |  |  |  |  |
| F2 | 0.000 | -5.368 | 0.000 | -3.621 | 0.000 | -9.614 |  |  |  |  |
| F4 |  |  |  |  | 0.000 | -5.015 |  |  |  |  |
| F6 | 0.000 | -5.804 | 0.005 | -2.790 | 0.000 | -6.547 |  |  |  |  |
| F8 | 0.000 | -5.429 | 0.000 | -4.571 | 0.000 | -7.090 |  |  |  |  |
| FT7 | 0.015 | -2.444 |  |  | 0.002 | -3.155 |  |  |  |  |
| FC5 | 0.000 | -6.010 | 0.000 | -3.959 | 0.000 | -4.876 |  |  |  |  |
| FC3 | 0.000 | -4.808 | 0.000 | -7.873 | 0.000 | -7.610 |  |  |  |  |
| FC1 |  |  |  |  | 0.000 | -3.733 |  |  |  |  |
| FCZ | 0.000 | -5.625 | 0.016 | -2.420 | 0.000 | -8.163 |  |  |  |  |
| FC2 | 0.000 | -5.649 | 0.000 | -4.705 | 0.000 | -8.567 |  |  |  |  |
| FC4 | 0.000 | -3.709 |  |  | 0.000 | -4.131 |  |  |  |  |
| FC6 | 0.000 | -7.728 |  |  | 0.000 | -5.164 |  |  |  |  |
| FT8 | 0.000 | -7.129 | 0.000 | -4.295 | 0.000 | -5.418 |  |  |  |  |
| T7 | 0.000 | -4.320 |  |  |  |  |  |  | 0.031 | -2.254 |
| C5 | 0.000 | -5.678 | 0.002 | -3.170 | 0.000 | -4.451 |  |  |  |  |
| C3 | 0.000 | -6.132 | 0.000 | -3.541 | 0.000 | -6.628 |  |  |  |  |
| C1 | 0.000 | -3.556 |  |  | 0.015 | -2.445 | 0.037 | -2.174 |  |  |
| CZ | 0.000 | -4.913 | 0.000 | -3.561 | 0.000 | -5.487 |  |  |  |  |
| C2 | 0.000 | -6.773 | 0.000 | -4.877 | 0.000 | -6.580 |  |  |  |  |
| C4 | 0.000 | -5.058 |  |  |  |  |  |  |  |  |
| C6 | 0.000 | -4.428 | 0.002 | -3.156 | 0.000 | -4.477 |  |  |  |  |
| T8 | 0.000 | -5.333 | 0.000 | -8.362 | 0.000 | -6.932 |  |  |  |  |
| TP7 | 0.000 | -4.031 | 0.000 | -4.561 | 0.018 | -2.367 |  |  |  |  |
| CP5 |  |  | 0.005 | -2.818 | 0.002 | -3.061 |  |  |  |  |
| CP3 | 0.001 | -3.266 | 0.000 | -6.421 | 0.000 | -4.741 |  |  |  |  |
| CP1 |  |  | 0.000 | -4.679 |  |  |  |  |  |  |
| CP2 | 0.000 | -4.992 | 0.000 | -4.647 | 0.000 | -3.618 |  |  |  |  |
| CP4 |  |  | 0.000 | -4.875 |  |  |  |  |  |  |
| CP6 | 0.000 | -4.960 | 0.000 | -3.558 | 0.000 | 4.356- |  |  |  |  |
| TP8 | 0.000 | -5.555 | 0.001 | -3.361 | 0.000 | -4.499 |  |  |  |  |
| P5 | 0.000 | -7.488 | 0.003 | -2.988 | 0.000 | -5.665 |  |  |  |  |
| P3 | 0.000 | -6.478 | 0.000 | -5.540 | 0.000 | -4.736 |  |  |  |  |
| PZ | 0.000 | -4.084 | 0.000 | -4.416 | 0.000 | -7.168 |  |  |  |  |
| P4 |  |  | 0.024 | -2.258 | 0.004 | -2.910 |  |  |  |  |
| P6 | 0.000 | -4.658 | 0.000 | -4.621 | 0.016 | -2.415 |  |  |  |  |
| P8 | 0.000 | -6.748 | 0.000 | -5.769 | 0.000 | -4.954 |  |  |  |  |
| PO7 | 0.000 | -6.173 | 0.000 | -4.825 | 0.004 | -2.871 |  |  |  |  |
| PO5 | 0.000 | -9.409 | 0.000 | -3.554 | 0.002 | -3.137 |  |  |  |  |
| PO3 | 0.000 | -7.104 | 0.000 | -3.544 | 0.000 | -3.956 |  |  |  |  |
| POZ | 0.000 | -6.725 | 0.000 | -4.727 | 0.006 | -2.763 |  |  |  |  |
| PO4 | 0.000 | -8.850 | 0.000 | -4.957 | 0.000 | -6.033 |  |  |  |  |
| PO6 | 0.000 | -4.131 | 0.000 | -3.741 | 0.000 | -4.459 |  |  |  |  |
| PO8 | 0.000 | -6.725 | 0.000 | -4.298 | 0.000 | -3.990 | 0.041 | -2.124 |  |  |
| O1 | 0.000 | -6.231 | 0.000 | -4.416 | 0.000 | -6.623 |  |  |  |  |
| OZ | 0.000 | -4.701 | 0.001 | -3.438 | 0.000 | -6.439 |  |  |  |  |
| O2 | 0.000 | -4.057 |  |  | 0.000 | -4.010 |  |  |  |  |

**Supplementary table2 P and T value of α band**

|  | Left-hand MI | | Right-hand MI | | Feet MI | | EO | | EC | |
| --- | --- | --- | --- | --- | --- | --- | --- | --- | --- | --- |
|  | P value | T value | P value | T value | P value | T value | P value | T value | P value | T value |
| FP1 | 0.000 | -4.599 | 0.040 | -2.057 | 0.005 | -2.796 |  |  |  | -2.026 |
| FPZ | 0.000 | -5.003 | 0.000 | -5.425 | 0.000 | -5.459 |  |  | 0.044 | -2.095 |
| FP2 | 0.031 | -2.168 |  |  | 0.031 | -2.158 |  |  | 0.033 | -2.228 |
| AF3 | 0.000 | -3.649 |  |  | 0.032 | -2.145 |  |  | 0.003 | -3.172 |
| AF4 | 0.000 | -3.811 |  |  | 0.001 | -3.419 |  |  |  |  |
| F7 | 0.009 | -2.637 |  |  |  |  |  |  |  |  |
| F5 | 0.000 | -3.911 |  |  | 0.014 | -2.455 |  |  | 0.002 | -3.319 |
| F3 | 0.012 | -2.530 | 0.000 | -4.857 | 0.000 | -4.637 |  |  | 0.002 | -3.320 |
| F1 |  |  |  |  | 0.003 | -3.022 |  |  | 0.003 | -3.223 |
| FZ |  |  | 0.000 | -3.545 |  |  |  |  | 0.020 | -2.440 |
| F2 | 0.002 | -3.154 | 0.000 | -3.772 | 0.000 | -4.961 |  |  | 0.030 | -2.260 |
| F4 |  |  | 0.012 | -2.318 | 0.000 | -3.988 |  |  |  |  |
| F6 | 0.032 | -2.152 |  |  | 0.012 | -2.518 |  |  | 0.013 | -2.610 |
| F8 | 0.000 | -4.797 | 0.000 | -5.150 | 0.000 | -5.557 | 0.014 | -2.602 | 0.025 | -2.338 |
| FT7 |  |  | 0.002 | -3.034 | 0.015 | -2.450 |  |  |  |  |
| FC5 | 0.000 | -5.650 | 0.000 | -3.943 | 0.001 | -3.208 |  |  | 0.017 | -2.500 |
| FC3 | 0.000 | -5.788 | 0.001 | -3.350 | 0.038 | -2.083 |  |  | 0.016 | -2.546 |
| FC1 | 0.000 | -3.945 |  |  | 0.002 | -3.046 | 0.007 | -2.857 | 0.028 | -2.291 |
| FCZ |  |  |  |  |  |  | 0.018 | -2.497 | 0.045 | -2.085 |
| FC2 | 0.000 | -3.898 | 0.000 | -4.415 | 0.000 | -4.559 |  |  |  |  |
| FC4 |  |  |  |  |  |  | 0.039 | -2.143 | 0.018 | -2.494 |
| FC6 | 0.000 | -4.990 |  |  |  |  | 0.018 | -2.489 | 0.009 | -2.762 |
| FT8 | 0.000 | -7.878 | 0.000 | 7.188- | 0.001 | -3.432 | 0.001 | -3.532 | 0.002 | -3.290 |
| T7 | 0.000 | -5.756 | 0.014 | -2.476 |  |  | 0.001 | -3.631 |  |  |
| C5 | 0.000 | -4.101 | 0.000 | -4.739 |  |  | 0.026 | -2.334 |  |  |
| C3 |  |  |  |  |  |  | 0.001 | -3.823 | 0.000 | -3.960 |
| C1 |  |  | 0.014 | -2.473 |  |  | 0.000 | -3.931 | 0.002 | -3.319 |
| CZ |  |  |  |  |  |  | 0.049 | -2.041 |  |  |
| C2 | 0.000 | -5.010 | 0.000 | -5.443 |  |  | 0.002 | -3.328 | 0.009 | -2.781 |
| C4 | 0.000 | -5.961 | 0.001 | -3.324 |  |  | 0.006 | -2.964 | 0.001 | -3.723 |
| C6 | 0.031 | -2.161 | 0.012 | -2.523 |  |  |  |  |  |  |
| T8 | 0.000 | --5.742 | 0.000 | -5.626 | 0.002 | -3.181 |  |  |  |  |
| TP7 | 0.001 | -3.219 | 0.000 | -3.855 |  |  |  |  | 0.015 | -2.549 |
| CP5 |  |  |  |  | 0.000 | -5.021 |  |  | 0.010 | -2.716 |
| CP3 | 0.002 | -3.102 | 0.000 | -4.454 |  |  |  |  |  |  |
| CP1 |  |  |  |  | 0.000 | -3.631 |  |  |  |  |
| CP2 | 0.006 | -2.764 | 0.000 | -3.670 |  |  |  |  | 0.002 | -3.321 |
| CP4 | 0.001 | -3.210 |  |  |  |  |  |  | 0.004 | -3.044 |
| CP6 | 0.000 | -3.822 | 0.000 | -4.591 |  |  |  |  |  |  |
| TP8 | 0.004 | -2.865 | 0.001 | -3.425 | 0.004 | -2.927 |  |  |  |  |
| P7 |  |  | 0.000 | -4.015 | 0.030 | -2.177 |  |  |  |  |
| P5 |  |  | 0.001 | -3.232 |  |  |  |  | 0.029 | -2.283 |
| P3 | 0.000 | -4.655 | 0.016 | -2.420 |  |  | 0.018 | -2.478 |  |  |
| PZ | 0.001 | -3.347 | 0.000 | -4.163 |  |  |  |  | 0.026 | -2.325 |
| P4 | 0.011 | -2.559 | 0.000 | -3.681 |  |  | 0.011 | -2.701 | 0.014 | -2.601 |
| P6 | 0.000 | -3.531 | 0.003 | -2.942 | 0.000 | -3.702 | 0.005 | -3.041 |  |  |
| P8 | 0.000 | -5.679 | 0.000 | -4.180 |  |  |  |  |  |  |
| PO7 | 0.000 | -5.796 | 0.000 | -4.622 |  |  | 0.049 | -2.042 |  |  |
| PO5 | 0.000 | -3.531 |  |  |  |  | 0.007 | -2.862 | 0.018 | -2.480 |
| PO3 | 0.000 | -4.796 |  |  |  |  | 0.000 | -3.901 | 0.004 | -3.067 |
| POZ | 0.000 | -5.104 | 0.014 | -2.475 |  |  | 0.001 | -3.530 |  |  |
| PO4 | 0.000 | -3.924 | 0.000 | -4.875 |  |  |  |  |  |  |
| PO6 | 0.034 | -2.125 |  |  |  |  | 0.001 | -3.521 | 0.000 | -4.249 |
| PO8 | 0.000 | -3.803 | 0.047 | -1.988 |  |  | 0.000 | -3.971 | 0.001 | -3.531 |
| O1 |  |  | 0.007 | -2.692 |  |  |  |  |  |  |
| OZ | 0.005 | -2.808 | 0.032 | -2.148 |  |  | 0.002 | -3.374 | 0.025 | -2.346 |
| O2 | 0.001 | -3.298 |  |  |  |  | 0.021 | -2.414 | 0.003 | -3.250 |

**Supplementary table3 P and T value of β band**

|  | Left-hand MI | | Right-hand MI | | Feet MI | | EO | | EC | |
| --- | --- | --- | --- | --- | --- | --- | --- | --- | --- | --- |
|  | P value | T value | P value | T value | P value | T value | P value | T value | P value | T value |
| FP1 |  |  | 0.015 | 2.441 | 0.000 | 4.602 |  |  | 0.007 | 2.850 |
| FPZ | 0.000 | 4.148 |  |  | 0.027 | 2.217 |  |  | 0.012 | 2.667 |
| FP2 | 0.007 | 2.699 |  |  | 0.000 | 3.830 | 0.019 | 2.465 | 0.011 | 2.673 |
| AF3 | 0.001 | 3.258 | 0.001 | 3.191 | 0.000 | 6.652 | 0.022 | 2.407 | 0.026 | 2.330 |
| AF4 | 0.002 | 3.036 | 0.001 | 3.395 | 0.000 | 3.835 |  |  |  |  |
| F7 |  |  | 0.022 | 2.296 |  |  | 0.037 | 2.175 | 0.008 | 2.818 |
| F5 | 0.000 | 3.789 | 0.000 | 5.366 | 0.002 | 3.101 |  |  |  |  |
| F3 | 0.000 | 6.311 |  |  |  |  |  |  | 0.019 | 2.463 |
| F1 | 0.006 | 2.765 |  |  |  |  |  |  | 0.000 | 4.003 |
| FZ | 0.000 | 5.658 | 0.005 | 2.786 | 0.000 | 4.578 |  |  | 0.039 | 2.146 |
| F2 | 0.003 | 3.000 | 0.005 | 2.823 | 0.001 | 3.300 |  |  | 0.048 | 2.053 |
| F4 | 0.003 | 2.955 | 0.005 | 2.808 |  |  | 0.027 | 2.319 | 0.017 | 2.500 |
| F6 | 0.000 | 4.725 | 0.000 | 4.272 | 0.000 | 5.784 | 0.047 | 2.061 | 0.001 | 3.503 |
| F8 | 0.000 | 3.634 |  |  | 0.021 | 2.312 |  |  | 0.000 | 3.895 |
| FT7 |  |  | 0.004 | 2.915 |  |  |  |  | 0.042 | 2.117 |
| FC5 | 0.000 | 3.965 | 0.000 | 3.689 | 0.003 | 2.974 | 0.009 | 2.770 | 0.000 | 4.124 |
| FC3 | 0.013 | 2.484 | 0.018 | 2.367 |  |  | 0.029 | 2.274 | 0.006 | 2.962 |
| FC1 | 0.002 | 3.052 |  |  | 0.008 | 2.653 |  |  | 0.009 | 2.788 |
| FCZ | 0.000 | 3.787 | 0.002 | 3.090 | 0.008 | 2.679 |  |  | 0.013 | 2.612 |
| FC2 | 0.000 | 5.152 | 0.000 | 3.554 |  |  |  |  | 0.038 | 2.159 |
| FC4 | 0.036 | 2.102 | 0.002 | 3.125 |  |  |  |  | 0.047 | 2.063 |
| FC6 | 0.000 | 3.529 | 0.005 | 2.809 |  |  |  |  |  |  |
| FT8 | 0.012 | 2.526 | 0.015 | 2.450 |  |  |  |  | 0.010 | 2.744 |
| T7 |  |  |  |  |  |  |  |  | 0.006 | 2.924 |
| C5 | 0.000 | 5.300 | 0.000 | 3.545 | 0.000 | 5.110 |  |  | 0.022 | 2.400 |
| C3 | 0.017 | 2.395 | 0.000 | 4.450 |  |  |  |  | 0.009 | 2.776 |
| C1 | 0.002 | 3.063 |  |  |  |  |  |  | 0.019 | 2.452 |
| CZ |  |  | 0.002 | 3.043 | 0.000 | 4.268 |  |  |  |  |
| C2 | 0.032 | 2.147 | 0.002 | 3.100 | 0.006 | 2.774 |  |  | 0.023 | 2.381 |
| C4 |  |  |  |  | 0.000 | 4.202 |  |  | 0.006 | 2.930 |
| C6 | 0.000 | 4.098 | 0.000 | 4.084 | 0.000 | 6.821 |  |  |  |  |
| T8 | 0.000 | 5.283 |  |  | 0.001 | 3.305 |  |  |  |  |
| TP7 |  |  |  |  |  |  |  |  | 0.003 | 3.203 |
| CP5 |  |  |  |  | 0.000 | 5.315 | 0.043 | 2.097 |  |  |
| CP3 | 0.000 | 4.530 |  |  | 0.000 | 3.826 |  |  |  |  |
| CP1 |  |  | 0.000 | 4.667 | 0.000 | 4.127 |  |  | 0.046 | 2.073 |
| CP2 |  |  |  |  | 0.001 | 3.412 |  |  |  |  |
| CP4 |  |  |  |  | 0.000 | 3.723 |  |  | 0.003 | 3.178 |
| CP6 | 0.000 | 4.421 | 0.001 | 3.254 | 0.000 | 4.374 |  |  |  |  |
| TP8 | 0.003 | 3.028 |  |  | 0.001 | 3.208 |  |  | 0.008 | 2.800 |
| P7 | 0.000 | 4.201 |  |  | 0.000 | 4.021 |  |  |  |  |
| P5 | 0.007 | 2.715 | 0.005 | 2.795 | 0.000 | 5.471 |  |  | 0.000 | 4.306 |
| P3 | 0.034 | 2.120 |  |  |  |  |  |  | 0.012 | 2.665 |
| PZ |  |  | 0.000 | 3.778 | 0.000 | 3.712 |  |  | 0.008 | 2.809 |
| P4 | 0.002 | 3.163 |  |  | 0.000 | 4.926 |  |  | 0.012 | 2.660 |
| P6 |  |  | 0.000 | 7.464 |  |  |  |  | 0.011 | 2.681 |
| P8 |  |  | 0.002 | 3.090 | 0.036 | 2.105 |  |  |  |  |
| PO7 | 0.019 | 2.359 |  |  | 0.000 | 4.644 |  |  |  |  |
| PO5 | 0.037 | 2.095 | 0.003 | 2.942 | 0.008 | 2.673 |  |  |  |  |
| PO3 |  |  | 0.000 | 3.813 | 0.003 | 2.965 |  |  | 0.001 | 3.705 |
| POZ |  |  |  |  |  |  |  |  | 0.017 | 2.509 |
| PO4 | 0.013 | 2.486 | 0.001 | 3.243 | 0.001 | 3.372 |  |  |  |  |
| PO6 | 0.001 | 3.320 | 0.000 | 3.505 | 0.005 | 2.793 |  |  | 0.030 | 2.267 |
| PO8 |  |  |  |  | 0.012 | 2.516 |  |  | 0.008 | 2.819 |
| O1 | 0.001 | 3.258 | 0.000 | 6.831 | 0.000 | 3.928 |  |  |  |  |
| OZ | 0.010 | 2.584 | 0.002 | 3.152 | 0.010 | 2.585 |  |  |  |  |
| O2 |  |  |  |  |  |  |  |  |  |  |

**Supplementary table4.** **Channels with differences after CWT and MST feature extraction**

| MI | Frequency band | CWT | MST |
| --- | --- | --- | --- |
| Left-hand MI | θ | 21 | 50 |
|  | α | 28 | 43 |
|  | β | 60 | 39 |
| Right-hand MI | θ | 35 | 42 |
|  | α | 53 | 37 |
|  | β | 60 | 34 |
| Feet MI | θ | 34 | 50 |
|  | α | 38 | 25 |
|  | β | 59 | 39 |

**Supplementary table5. The results of the normality test of θ band**

| Channel | Left-hand MI | | | | Right-hand MI | | | | Feet MI | | | | EO | | | | EC | | | |
| --- | --- | --- | --- | --- | --- | --- | --- | --- | --- | --- | --- | --- | --- | --- | --- | --- | --- | --- | --- | --- |
|  | PWP | | PWN | | PWP | | PWN | | PWP | | PWN | | PWP | | PWN | | PWP | | PWN | |
|  | Z | P | Z | P | Z | P | Z | P | Z | P | Z | P | Z | P | Z | P | Z | P | Z | P |
| FP1 | .261 | .052 | .207 | .167 | .174 | .200 | .262 | .076 | .237 | .118 | .222 | .200 | .187 | .137 | .201 | .200 | .164 | .200 | .107 | .200 |
| FPZ | .261 | .052 | .207 | .167 | .128 | .200 | .278 | .043 | .192 | .200 | .251 | .200 | .170 | .200 | .217 | .200 | .082 | .200 | .191 | .082 |
| FP2 | .228 | .151 | .220 | .114 | .245 | .046 | .207 | .051 | .172 | .200 | .188 | .200 | .171 | .200 | .228 | .152 | .150 | .200 | .143 | .200 |
| AF3 | .197 | .096 | .261 | .052 | .187 | .139 | .262 | .076 | .221 | .181 | .257 | .200 | .141 | .200 | .201 | .083 | .167 | .198 | .178 | .138 |
| AF4 | .187 | .137 | .206 | .168 | .217 | .125 | .283 | .037 | .234 | .128 | .268 | .200 | .097 | .200 | .201 | .082 | .114 | .200 | .143 | .200 |
| F7 | .170 | .200 | .246 | .042 | .242 | .051 | .233 | .171 | .263 | .049 | .318 | .109 | .129 | .200 | .163 | .200 | .126 | .200 | .114 | .200 |
| F5 | .171 | .200 | .188 | .200 | .212 | .142 | .236 | .161 | .206 | .200 | .239 | .200 | .215 | .047 | .197 | .096 | .187 | .096 | .128 | .200 |
| F3 | .141 | .200 | .217 | .125 | .241 | .052 | .158 | .200 | .269 | .039 | .322 | .098 | .131 | .200 | .187 | .137 | .121 | .200 | .157 | .200 |
| F1 | .212 | .068 | .253 | .032 | .246 | .043 | .162 | .200 | .242 | .101 | .310 | .132 | .199 | .091 | .170 | .200 | .129 | .200 | .093 | .200 |
| FZ | .206 | .085 | .229 | .112 | .192 | .200 | .222 | .034 | .255 | .065 | .334 | .071 | .205 | .070 | .171 | .200 | .138 | .200 | .152 | .200 |
| F2 | .143 | .200 | .189 | .200 | .252 | .034 | .293 | .025 | .232 | .135 | .307 | .139 | .165 | .200 | .253 | .069 | .134 | .200 | .122 | .200 |
| F4 | .150 | .200 | .190 | .200 | .216 | .129 | .177 | .200 | .180 | .200 | .245 | .200 | .109 | .200 | .212 | .200 | .167 | .200 | .093 | .200 |
| F6 | .102 | .200 | .180 | .200 | .200 | .200 | .143 | .200 | .236 | .120 | .242 | .200 | .150 | .200 | .194 | .200 | .172 | .166 | .159 | .200 |
| F8 | .200 | .159 | .234 | .068 | .263 | .021 | .178 | .138 | .203 | .200 | .252 | .200 | .155 | .200 | .201 | .083 | .170 | .180 | .082 | .200 |
| FT7 | .147 | .200 | .300 | .099 | .228 | .085 | .261 | .052 | .241 | .104 | .255 | .200 | .118 | .200 | .201 | .082 | .173 | .165 | .166 | .200 |
| FC5 | .164 | .200 | .233 | .071 | .246 | .043 | .215 | .133 | .167 | .200 | .185 | .200 | .134 | .200 | .163 | .200 | .126 | .200 | .168 | .192 |
| FC3 | .082 | .200 | .186 | .200 | .251 | .035 | .225 | .200 | .164 | .200 | .252 | .200 | .134 | .200 | .212 | .200 | .112 | .200 | .178 | .137 |
| FC1 | .150 | .200 | .178 | .200 | .171 | .200 | .178 | .138 | .218 | .196 | .241 | .200 | .178 | .190 | .166 | .200 | .159 | .200 | .124 | .200 |
| FCZ | .191 | .200 | .180 | .200 | .158 | .200 | .261 | .052 | .254 | .067 | .185 | .200 | .164 | .200 | .236 | .120 | .107 | .200 | .116 | .200 |
| FC2 | .145 | .200 | .211 | .146 | .162 | .200 | .215 | .133 | .212 | .200 | .243 | .200 | .152 | .200 | .256 | .061 | .126 | .200 | .180 | .129 |
| FC4 | .220 | .188 | .211 | .147 | .222 | .034 | .228 | .085 | .248 | .082 | .244 | .200 | .151 | .200 | .236 | .120 | .098 | .200 | .107 | .200 |
| FC6 | .259 | .055 | .250 | .037 | .129 | .200 | .246 | .043 | .210 | .200 | .286 | .200 | .131 | .200 | .237 | .117 | .096 | .200 | .178 | .138 |
| FT8 | .160 | .200 | .209 | .153 | .200 | .159 | .212 | .142 | .203 | .200 | .214 | .200 | .108 | .200 | .274 | .032 | .134 | .200 | .135 | .200 |
| T7 | .173 | .200 | .200 | .159 | .229 | .083 | .267 | .064 | .214 | .200 | .210 | .200 | .127 | .200 | .197 | .096 | .139 | .200 | .141 | .200 |
| C5 | .188 | .200 | .204 | .074 | .259 | .025 | .212 | .068 | .281 | .151 | .224 | .200 | .172 | .200 | .265 | .045 | .119 | .200 | .115 | .200 |
| C3 | .207 | .051 | .220 | .112 | .212 | .068 | .254 | .097 | .254 | .066 | .243 | .200 | .102 | .200 | .250 | .077 | .130 | .200 | .191 | .082 |
| C1 | .262 | .076 | .178 | .138 | .206 | .085 | .199 | .200 | .242 | .099 | .217 | .200 | .143 | .200 | .192 | .200 | .132 | .200 | .134 | .200 |
| CZ | .213 | .200 | .261 | .052 | .187 | .137 | .209 | .200 | .190 | .200 | .254 | .200 | .185 | .145 | .207 | .165 | .148 | .200 | .128 | .200 |
| C2 | .186 | .200 | .203 | .183 | .228 | .084 | .217 | .200 | .208 | .200 | .196 | .200 | .180 | .176 | .190 | .200 | .136 | .200 | .137 | .200 |
| C4 | .152 | .200 | .218 | .119 | .139 | .200 | .279 | .042 | .224 | .170 | .223 | .200 | .180 | .172 | .286 | .020 | .165 | .200 | .116 | .200 |
| C6 | .130 | .200 | .179 | .200 | .206 | .171 | .224 | .200 | .232 | .136 | .212 | .200 | .262 | .076 | .196 | .200 | .176 | .144 | .106 | .200 |
| T8 | .161 | .200 | .226 | .092 | .219 | .115 | .192 | .200 | .207 | .200 | .249 | .200 | .206 | .068 | .219 | .192 | .112 | .200 | .153 | .200 |
| TP7 | .151 | .200 | .191 | .200 | .209 | .153 | .205 | .177 | .165 | .200 | .194 | .200 | .193 | .114 | .218 | .195 | .154 | .200 | .103 | .200 |
| CP5 | .241 | .104 | .224 | .097 | .242 | .051 | .206 | .053 | .229 | .147 | .250 | .200 | .190 | .125 | .262 | .050 | .164 | .200 | .164 | .200 |
| CP3 | .200 | .159 | .241 | .052 | .222 | .106 | .248 | .118 | .246 | .089 | .274 | .200 | .129 | .200 | .197 | .096 | .109 | .200 | .143 | .200 |
| CP1 | .158 | .200 | .251 | .035 | .246 | .043 | .214 | .200 | .271 | .035 | .281 | .200 | .111 | .200 | .187 | .137 | .131 | .200 | .126 | .200 |
| CP2 | .282 | .023 | .215 | .133 | .215 | .133 | .206 | .171 | .204 | .200 | .215 | .200 | .140 | .200 | .160 | .200 | .110 | .200 | .140 | .200 |
| CP4 | .130 | .200 | .256 | .029 | .189 | .200 | .219 | .115 | .229 | .112 | .299 | .165 | .185 | .144 | .262 | .050 | .167 | .200 | .141 | .200 |
| CP6 | .218 | .194 | .229 | .112 | .183 | .200 | .209 | .153 | .239 | .112 | .276 | .200 | .186 | .141 | .197 | .096 | .131 | .200 | .147 | .200 |
| TP8 | .226 | .159 | .281 | .151 | .207 | .051 | .281 | .039 | .253 | .069 | .305 | .145 | .124 | .200 | .187 | .137 | .104 | .200 | .154 | .200 |
| P7 | .207 | .051 | .178 | .138 | .172 | .200 | .178 | .200 | .250 | .077 | .312 | .124 | .080 | .200 | .258 | .059 | .172 | .167 | .102 | .200 |
| P5 | .262 | .076 | .261 | .052 | .208 | .162 | .141 | .200 | .182 | .200 | .236 | .200 | .168 | .200 | .228 | .149 | .194 | .072 | .167 | .200 |
| P3 | .254 | .066 | .226 | .092 | .203 | .183 | .212 | .068 | .233 | .134 | .151 | .200 | .160 | .200 | .157 | .200 | .143 | .200 | .166 | .200 |
| PZ | .179 | .200 | .194 | .200 | .217 | .124 | .206 | .085 | .205 | .200 | .258 | .200 | .133 | .200 | .251 | .075 | .119 | .200 | .178 | .138 |
| P4 | .259 | .055 | .141 | .200 | .239 | .057 | .228 | .085 | .237 | .117 | .257 | .200 | .130 | .200 | .273 | .033 | .136 | .200 | .155 | .200 |
| P6 | .187 | .200 | .260 | .024 | .203 | .124 | .281 | .039 | .175 | .200 | .198 | .200 | .213 | .051 | .228 | .152 | .132 | .200 | .118 | .200 |
| P8 | .208 | .200 | .215 | .133 | .174 | .200 | .206 | .171 | .176 | .200 | .276 | .200 | .154 | .200 | .220 | .186 | .092 | .200 | .197 | .063 |
| PO7 | .172 | .200 | .228 | .085 | .128 | .200 | .219 | .115 | .224 | .167 | .241 | .200 | .120 | .200 | .180 | .200 | .095 | .200 | .179 | .130 |
| PO5 | .236 | .120 | .246 | .043 | .255 | .031 | .230 | .187 | .249 | .079 | .192 | .200 | .140 | .200 | .239 | .109 | .125 | .200 | .099 | .200 |
| PO3 | .190 | .200 | .212 | .142 | .207 | .106 | .228 | .152 | .205 | .200 | .256 | .200 | .130 | .200 | .265 | .046 | .108 | .200 | .141 | .200 |
| POZ | .218 | .197 | .259 | .026 | .214 | .083 | .282 | .038 | .236 | .122 | .226 | .200 | .117 | .200 | .253 | .070 | .152 | .200 | .124 | .200 |
| PO4 | .169 | .200 | .217 | .124 | .230 | .043 | .197 | .096 | .206 | .200 | .273 | .200 | .116 | .200 | .226 | .158 | .126 | .200 | .136 | .200 |
| PO6 | .220 | .114 | .180 | .200 | .219 | .067 | .245 | .126 | .199 | .200 | .199 | .200 | .130 | .200 | .271 | .036 | .151 | .200 | .194 | .073 |
| PO8 | .261 | .052 | .207 | .165 | .198 | .143 | .210 | .200 | .217 | .199 | .206 | .200 | .201 | .083 | .228 | .152 | .125 | .200 | .181 | .124 |
| O1 | .180 | .200 | .186 | .140 | .146 | .200 | .249 | .115 | .261 | .052 | .222 | .200 | .201 | .082 | .267 | .041 | .162 | .200 | .186 | .100 |
| OZ | .120 | .200 | .153 | .200 | .218 | .069 | .212 | .200 | .237 | .118 | .273 | .200 | .163 | .200 | .243 | .098 | .133 | .200 | .126 | .200 |
| O2 | .261 | .052 | .207 | .167 | .174 | .200 | .262 | .076 | .237 | .118 | .222 | .200 | .187 | .137 | .201 | .200 | .164 | .200 | .107 | .200 |

**Supplementary table6. The results of the normality test of α band**

| Channel | Left-hand MI | | | | Right-hand MI | | | | Feet MI | | | | EO | | | | EC | | | |
| --- | --- | --- | --- | --- | --- | --- | --- | --- | --- | --- | --- | --- | --- | --- | --- | --- | --- | --- | --- | --- |
|  | PWP | | PWN | | PWP | | PWN | | PWP | | PWN | | PWP | | PWN | | PWP | | PWN | |
|  | Z | P | Z | P | Z | P | Z | P | Z | P | Z | P | Z | P | Z | P | Z | P | Z | P |
| FP1 | .188 | .200 | .282 | .061 | .178 | .138 | .150 | .200 | .262 | .076 | .203 | .124 | .212 | .200 | .260 | .200 | .212 | .068 | .151 | .200 |
| FPZ | .169 | .200 | .203 | .200 | .261 | .052 | .143 | .200 | .147 | .200 | .174 | .200 | .208 | .200 | .281 | .151 | .206 | .085 | .197 | .096 |
| FP2 | .221 | .108 | .287 | .051 | .215 | .133 | .178 | .138 | .176 | .200 | .128 | .200 | .249 | .200 | .272 | .188 | .143 | .200 | .187 | .200 |
| AF3 | .194 | .200 | .235 | .066 | .248 | .200 | .261 | .052 | .126 | .200 | .218 | .071 | .139 | .200 | .300 | .099 | .170 | .200 | .213 | .111 |
| AF4 | .217 | .126 | .240 | .194 | .248 | .200 | .215 | .133 | .177 | .200 | .194 | .162 | .146 | .200 | .206 | .200 | .212 | .067 | .213 | .051 |
| F7 | .180 | .200 | .225 | .200 | .298 | .060 | .228 | .085 | .247 | .060 | .201 | .130 | .293 | .069 | .227 | .200 | .212 | .067 | .138 | .200 |
| F5 | .250 | .037 | .236 | .200 | .194 | .200 | .178 | .157 | .226 | .121 | .161 | .200 | .161 | .200 | .211 | .200 | .184 | .185 | .138 | .200 |
| F3 | .130 | .200 | .238 | .200 | .134 | .200 | .210 | .045 | .211 | .185 | .186 | .140 | .208 | .200 | .288 | .130 | .178 | .200 | .190 | .200 |
| F1 | .232 | .074 | .228 | .200 | .177 | .200 | .208 | .048 | .177 | .200 | .219 | .066 | .251 | .200 | .200 | .200 | .170 | .200 | .229 | .112 |
| FZ | .212 | .141 | .239 | .200 | .258 | .177 | .177 | .165 | .258 | .039 | .147 | .200 | .196 | .200 | .283 | .145 | .204 | .094 | .281 | .151 |
| F2 | .170 | .200 | .186 | .200 | .229 | .200 | .195 | .086 | .167 | .200 | .176 | .200 | .172 | .200 | .251 | .052 | .219 | .052 | .272 | .188 |
| F4 | .202 | .080 | .294 | .041 | .308 | .044 | .114 | .200 | .134 | .200 | .126 | .200 | .199 | .200 | .283 | .059 | .129 | .200 | .300 | .099 |
| F6 | .198 | .200 | .245 | .172 | .282 | .097 | .194 | .089 | .124 | .200 | .209 | .194 | .165 | .200 | .259 | .055 | .199 | .115 | .262 | .076 |
| F8 | .212 | .200 | .227 | .200 | .187 | .200 | .199 | .072 | .262 | .033 | .207 | .106 | .161 | .200 | .330 | .040 | .147 | .200 | .226 | .068 |
| FT7 | .208 | .200 | .205 | .200 | .243 | .200 | .164 | .200 | .186 | .200 | .154 | .200 | .291 | .074 | .192 | .200 | .206 | .088 | .151 | .200 |
| FC5 | .147 | .200 | .277 | .072 | .180 | .200 | .187 | .115 | .241 | .075 | .182 | .200 | .224 | .200 | .242 | .200 | .213 | .065 | .227 | .066 |
| FC3 | .180 | .200 | .203 | .200 | .182 | .200 | .207 | .051 | .186 | .140 | .151 | .200 | .237 | .200 | .336 | .033 | .185 | .179 | .146 | .200 |
| FC1 | .120 | .200 | .270 | .087 | .143 | .200 | .158 | .200 | .190 | .200 | .168 | .200 | .194 | .200 | .250 | .053 | .209 | .077 | .197 | .096 |
| FCZ | .194 | .200 | .259 | .122 | .295 | .067 | .228 | .152 | .228 | .113 | .163 | .200 | .280 | .102 | .298 | .103 | .217 | .056 | .200 | .163 |
| FC2 | .134 | .200 | .190 | .200 | .192 | .200 | .178 | .158 | .215 | .166 | .134 | .200 | .235 | .200 | .281 | .152 | .251 | .052 | .141 | .200 |
| FC4 | .261 | .052 | .266 | .101 | .325 | .025 | .135 | .200 | .240 | .075 | .153 | .200 | .261 | .052 | .270 | .196 | .114 | .200 | .207 | .165 |
| FC6 | .210 | .045 | .182 | .200 | .185 | .200 | .173 | .187 | .181 | .200 | .172 | .200 | .243 | .200 | .212 | .200 | .150 | .200 | .189 | .200 |
| FT8 | .208 | .048 | .259 | .123 | .313 | .037 | .194 | .200 | .226 | .123 | .186 | .140 | .245 | .200 | .208 | .200 | .205 | .090 | .215 | .133 |
| T7 | .147 | .200 | .164 | .200 | .214 | .200 | .134 | .200 | .209 | .194 | .221 | .062 | .271 | .130 | .249 | .200 | .187 | .137 | .200 | .161 |
| C5 | .164 | .200 | .283 | .059 | .183 | .200 | .197 | .079 | .165 | .200 | .176 | .200 | .298 | .059 | .189 | .200 | .164 | .200 | .236 | .046 |
| C3 | .082 | .200 | .225 | .200 | .164 | .200 | .159 | .200 | .194 | .200 | .187 | .199 | .290 | .077 | .288 | .130 | .283 | .059 | .199 | .168 |
| C1 | .150 | .200 | .193 | .200 | .214 | .200 | .209 | .046 | .189 | .200 | .189 | .190 | .297 | .062 | .205 | .177 | .199 | .113 | .183 | .200 |
| CZ | .167 | .198 | .130 | .200 | .210 | .200 | .152 | .200 | .199 | .200 | .144 | .200 | .212 | .200 | .178 | .200 | .155 | .200 | .171 | .200 |
| C2 | .130 | .200 | .210 | .200 | .191 | .200 | .214 | .038 | .171 | .200 | .259 | .055 | .203 | .200 | .344 | .025 | .185 | .178 | .300 | .099 |
| C4 | .107 | .200 | .265 | .105 | .321 | .028 | .220 | .028 | .208 | .200 | .207 | .106 | .299 | .058 | .266 | .200 | .111 | .200 | .224 | .075 |
| C6 | .199 | .200 | .239 | .200 | .093 | .200 | .186 | .140 | .232 | .100 | .214 | .083 | .250 | .200 | .181 | .200 | .188 | .159 | .136 | .200 |
| T8 | .166 | .200 | .196 | .200 | .244 | .200 | .153 | .200 | .219 | .146 | .230 | .043 | .160 | .200 | .212 | .200 | .208 | .080 | .246 | .030 |
| TP7 | .257 | .028 | .272 | .082 | .245 | .200 | .141 | .200 | .201 | .200 | .219 | .067 | .305 | .048 | .279 | .158 | .212 | .069 | .166 | .200 |
| CP5 | .199 | .200 | .179 | .200 | .311 | .039 | .179 | .181 | .127 | .200 | .198 | .143 | .159 | .200 | .253 | .200 | .207 | .051 | .174 | .200 |
| CP3 | .221 | .108 | .227 | .200 | .258 | .175 | .162 | .200 | .200 | .200 | .146 | .200 | .180 | .200 | .143 | .200 | .173 | .200 | .228 | .152 |
| CP1 | .196 | .200 | .128 | .200 | .203 | .200 | .188 | .134 | .179 | .200 | .218 | .069 | .219 | .200 | .201 | .200 | .202 | .099 | .201 | .083 |
| CP2 | .235 | .066 | .227 | .200 | .191 | .082 | .155 | .200 | .220 | .144 | .147 | .200 | .162 | .200 | .331 | .038 | .201 | .106 | .201 | .082 |
| CP4 | .190 | .200 | .283 | .059 | .266 | .144 | .174 | .179 | .227 | .119 | .259 | .055 | .225 | .200 | .246 | .200 | .230 | .031 | .163 | .200 |
| CP6 | .163 | .200 | .178 | .200 | .230 | .200 | .214 | .038 | .266 | .029 | .227 | .050 | .184 | .200 | .228 | .200 | .262 | .076 | .197 | .096 |
| TP8 | .179 | .200 | .150 | .200 | .147 | .200 | .143 | .200 | .176 | .200 | .147 | .200 | .190 | .200 | .250 | .200 | .219 | .051 | .187 | .137 |
| P7 | .168 | .200 | .189 | .200 | .261 | .052 | .178 | .138 | .246 | .061 | .176 | .200 | .182 | .200 | .212 | .200 | .125 | .200 | .191 | .082 |
| P5 | .189 | .200 | .244 | .179 | .257 | .180 | .261 | .052 | .121 | .200 | .126 | .200 | .215 | .200 | .208 | .200 | .182 | .194 | .212 | .068 |
| P3 | .222 | .104 | .223 | .200 | .269 | .136 | .215 | .133 | .164 | .200 | .192 | .174 | .185 | .200 | .249 | .200 | .185 | .180 | .206 | .085 |
| PZ | .153 | .200 | .250 | .149 | .217 | .200 | .228 | .085 | .268 | .027 | .236 | .033 | .189 | .200 | .281 | .149 | .222 | .046 | .218 | .093 |
| P4 | .256 | .029 | .244 | .178 | .208 | .200 | .246 | .043 | .273 | .021 | .138 | .200 | .211 | .200 | .226 | .200 | .169 | .200 | .217 | .200 |
| P6 | .130 | .200 | .157 | .200 | .171 | .200 | .212 | .142 | .248 | .058 | .215 | .078 | .223 | .200 | .252 | .200 | .209 | .077 | .172 | .200 |
| P8 | .107 | .200 | .197 | .200 | .289 | .079 | .217 | .124 | .127 | .200 | .221 | .062 | .231 | .200 | .206 | .068 | .228 | .152 | .189 | .200 |
| PO7 | .191 | .082 | .202 | .200 | .161 | .200 | .180 | .200 | .178 | .200 | .189 | .186 | .311 | .039 | .212 | .200 | .136 | .200 | .251 | .024 |
| PO5 | .143 | .200 | .255 | .135 | .239 | .200 | .173 | .186 | .235 | .090 | .226 | .051 | .253 | .194 | .231 | .200 | .196 | .126 | .217 | .095 |
| PO3 | .178 | .138 | .237 | .200 | .274 | .120 | .186 | .140 | .203 | .200 | .207 | .107 | .113 | .200 | .264 | .200 | .147 | .200 | .201 | .104 |
| POZ | .120 | .200 | .290 | .046 | .155 | .200 | .153 | .200 | .215 | .164 | .136 | .200 | .235 | .200 | .299 | .101 | .116 | .200 | .158 | .200 |
| PO4 | .194 | .200 | .228 | .152 | .209 | .200 | .141 | .200 | .259 | .055 | .178 | .200 | .191 | .200 | .212 | .200 | .202 | .152 | .237 | .023 |
| PO6 | .134 | .200 | .235 | .200 | .242 | .200 | .150 | .200 | .236 | .088 | .261 | .052 | .273 | .123 | .208 | .200 | .138 | .200 | .173 | .165 |
| PO8 | .261 | .052 | .240 | .194 | .149 | .200 | .208 | .049 | .183 | .200 | .199 | .139 | .191 | .082 | .323 | .050 | .159 | .200 | .150 | .200 |
| O1 | .282 | .061 | .213 | .111 | .298 | .061 | .206 | .053 | .184 | .200 | .210 | .096 | .253 | .197 | .221 | .200 | .226 | .067 | .110 | .200 |
| OZ | .188 | .200 | .151 | .200 | .234 | .200 | .146 | .200 | .251 | .052 | .196 | .150 | .119 | .200 | .303 | .091 | .210 | .119 | .164 | .200 |
| O2 | .256 | .029 | .216 | .200 | .239 | .057 | .153 | .200 | .234 | .093 | .259 | .055 | .154 | .200 | .260 | .200 | .157 | .200 | .154 | .200 |

**Supplementary table7. The results of the normality test of β band**

| Channel | Left-hand MI | | | | Right-hand MI | | | | Feet MI | | | | EO | | | | EC | | | |
| --- | --- | --- | --- | --- | --- | --- | --- | --- | --- | --- | --- | --- | --- | --- | --- | --- | --- | --- | --- | --- |
|  | PWP | | PWN | | PWP | | PWN | | PWP | | PWN | | PWP | | PWN | | PWP | | PWN | |
|  | Z | P | Z | P | Z | P | Z | P | Z | P | Z | P | Z | P | Z | P | Z | P | Z | P |
| FP1 | .153 | .200 | .186 | .140 | .158 | .200 | .187 | .139 | .152 | .200 | .191 | .200 | .191 | .200 | .228 | .026 | .150 | .200 | .154 | .200 |
| FPZ | .112 | .200 | .153 | .200 | .162 | .200 | .215 | .046 | .159 | .200 | .191 | .082 | .204 | .142 | .229 | .112 | .102 | .200 | .212 | .068 |
| FP2 | .240 | .144 | .141 | .200 | .222 | .034 | .206 | .067 | .189 | .200 | .251 | .052 | .227 | .067 | .193 | .113 | .200 | .159 | .206 | .085 |
| AF3 | .180 | .200 | .179 | .181 | .129 | .200 | .178 | .138 | .205 | .177 | .229 | .112 | .156 | .200 | .228 | .152 | .212 | .068 | .143 | .200 |
| AF4 | .181 | .200 | .162 | .200 | .227 | .026 | .151 | .200 | .132 | .200 | .229 | .110 | .146 | .200 | .133 | .200 | .182 | .200 | .150 | .200 |
| F7 | .163 | .200 | .188 | .134 | .149 | .200 | .181 | .167 | .204 | .182 | .189 | .200 | .234 | .049 | .176 | .200 | .200 | .161 | .128 | .200 |
| F5 | .120 | .200 | .194 | .110 | .170 | .200 | .156 | .200 | .264 | .021 | .155 | .200 | .197 | .096 | .180 | .178 | .106 | .200 | .187 | .168 |
| F3 | .200 | .200 | .202 | .080 | .199 | .089 | .179 | .181 | .204 | .179 | .199 | .200 | .150 | .200 | .178 | .138 | .165 | .200 | .166 | .200 |
| F1 | .212 | .200 | .178 | .138 | .188 | .133 | .204 | .074 | .225 | .095 | .191 | .200 | .176 | .200 | .186 | .140 | .164 | .200 | .198 | .118 |
| FZ | .219 | .200 | .176 | .200 | .212 | .068 | .196 | .102 | .250 | .077 | .135 | .200 | .208 | .129 | .176 | .200 | .139 | .200 | .115 | .200 |
| F2 | .148 | .200 | .120 | .200 | .206 | .085 | .181 | .171 | .109 | .200 | .254 | .045 | .238 | .043 | .186 | .140 | .169 | .200 | .183 | .187 |
| F4 | .181 | .200 | .214 | .049 | .108 | .200 | .174 | .200 | .168 | .200 | .224 | .130 | .217 | .097 | .228 | .085 | .191 | .200 | .154 | .200 |
| F6 | .215 | .200 | .151 | .200 | .224 | .032 | .162 | .200 | .298 | .059 | .253 | .048 | .206 | .134 | .239 | .112 | .179 | .200 | .178 | .200 |
| F8 | .194 | .200 | .184 | .150 | .214 | .048 | .158 | .200 | .205 | .177 | .225 | .126 | .226 | .068 | .253 | .069 | .165 | .200 | .132 | .200 |
| FT7 | .163 | .200 | .188 | .200 | .222 | .034 | .154 | .200 | .105 | .200 | .222 | .135 | .132 | .200 | .250 | .077 | .138 | .200 | .214 | .064 |
| FC5 | .162 | .200 | .169 | .200 | .212 | .142 | .165 | .200 | .201 | .196 | .170 | .200 | .195 | .189 | .182 | .200 | .164 | .200 | .158 | .200 |
| FC3 | .228 | .197 | .148 | .200 | .215 | .046 | .183 | .155 | .249 | .038 | .267 | .027 | .189 | .200 | .233 | .134 | .246 | .030 | .164 | .200 |
| FC1 | .142 | .200 | .133 | .200 | .206 | .067 | .130 | .200 | .250 | .036 | .165 | .200 | .170 | .200 | .205 | .200 | .160 | .200 | .216 | .057 |
| FCZ | .225 | .200 | .224 | .097 | .208 | .063 | .117 | .200 | .168 | .200 | .232 | .100 | .180 | .200 | .221 | .036 | .133 | .200 | .151 | .200 |
| FC2 | .167 | .198 | .147 | .200 | .136 | .200 | .195 | .105 | .220 | .113 | .235 | .090 | .197 | .179 | .180 | .177 | .181 | .200 | .197 | .122 |
| FC4 | .177 | .200 | .137 | .200 | .154 | .200 | .171 | .200 | .132 | .200 | .191 | .082 | .192 | .200 | .222 | .035 | .247 | .029 | .150 | .200 |
| FC6 | .108 | .200 | .204 | .074 | .209 | .060 | .201 | .200 | .259 | .055 | .261 | .052 | .219 | .089 | .212 | .200 | .100 | .200 | .102 | .200 |
| FT8 | .158 | .200 | .186 | .143 | .207 | .165 | .217 | .200 | .199 | .200 | .258 | .039 | .158 | .200 | .208 | .200 | .208 | .128 | .200 | .159 |
| T7 | .206 | .085 | .191 | .120 | .173 | .200 | .228 | .152 | .158 | .200 | .250 | .053 | .172 | .200 | .249 | .200 | .162 | .200 | .173 | .200 |
| C5 | .150 | .200 | .134 | .200 | .182 | .163 | .151 | .200 | .193 | .200 | .151 | .200 | .202 | .149 | .139 | .200 | .153 | .200 | .196 | .127 |
| C3 | .210 | .045 | .171 | .200 | .261 | .052 | .187 | .200 | .207 | .051 | .215 | .133 | .228 | .085 | .196 | .182 | .203 | .148 | .151 | .200 |
| C1 | .208 | .048 | .164 | .200 | .200 | .085 | .213 | .111 | .200 | .159 | .207 | .165 | .178 | .138 | .201 | .083 | .123 | .200 | .187 | .200 |
| CZ | .149 | .200 | .184 | .152 | .177 | .195 | .261 | .052 | .225 | .094 | .186 | .140 | .261 | .052 | .196 | .182 | .133 | .200 | .213 | .111 |
| C2 | .199 | .200 | .143 | .200 | .122 | .200 | .130 | .200 | .223 | .102 | .245 | .065 | .210 | .120 | .159 | .200 | .187 | .200 | .191 | .082 |
| C4 | .200 | .159 | .218 | .040 | .261 | .052 | .107 | .200 | .131 | .200 | .253 | .047 | .171 | .200 | .200 | .163 | .141 | .200 | .125 | .200 |
| C6 | .183 | .200 | .197 | .096 | .163 | .200 | .191 | .082 | .215 | .132 | .233 | .072 | .246 | .043 | .197 | .096 | .148 | .200 | .228 | .152 |
| T8 | .192 | .200 | .225 | .029 | .205 | .070 | .143 | .200 | .191 | .200 | .227 | .118 | .250 | .026 | .211 | .055 | .177 | .200 | .201 | .083 |
| TP7 | .149 | .200 | .147 | .200 | .124 | .200 | .178 | .138 | .162 | .200 | .213 | .111 | .167 | .200 | .181 | .166 | .207 | .130 | .201 | .082 |
| CP5 | .204 | .200 | .197 | .098 | .149 | .200 | .191 | .082 | .229 | .083 | .210 | .191 | .143 | .200 | .212 | .142 | .188 | .200 | .163 | .200 |
| CP3 | .182 | .200 | .227 | .027 | .158 | .200 | .175 | .200 | .257 | .028 | .229 | .112 | .175 | .200 | .220 | .037 | .137 | .200 | .197 | .096 |
| CP1 | .159 | .200 | .210 | .045 | .099 | .200 | .114 | .200 | .211 | .147 | .217 | .156 | .143 | .200 | .184 | .152 | .222 | .078 | .187 | .137 |
| CP2 | .234 | .168 | .208 | .048 | .183 | .155 | .175 | .200 | .206 | .169 | .177 | .200 | .196 | .182 | .132 | .200 | .161 | .200 | .170 | .200 |
| CP4 | .181 | .200 | .187 | .200 | .121 | .200 | .246 | .043 | .206 | .171 | .160 | .200 | .159 | .200 | .227 | .027 | .215 | .104 | .171 | .200 |
| CP6 | .169 | .200 | .206 | .069 | .088 | .200 | .167 | .200 | .219 | .115 | .250 | .053 | .200 | .163 | .205 | .070 | .195 | .191 | .253 | .069 |
| TP8 | .126 | .200 | .161 | .200 | .207 | .051 | .133 | .200 | .209 | .153 | .117 | .200 | .172 | .200 | .183 | .158 | .171 | .200 | .150 | .200 |
| P7 | .189 | .200 | .187 | .137 | .117 | .200 | .141 | .200 | .202 | .190 | .211 | .200 | .225 | .070 | .186 | .141 | .213 | .110 | .102 | .200 |
| P5 | .135 | .200 | .170 | .200 | .184 | .148 | .212 | .068 | .236 | .063 | .222 | .200 | .144 | .200 | .212 | .068 | .175 | .200 | .200 | .159 |
| P3 | .152 | .200 | .171 | .200 | .137 | .200 | .206 | .085 | .223 | .101 | .251 | .200 | .250 | .053 | .206 | .085 | .211 | .115 | .163 | .200 |
| PZ | .277 | .044 | .153 | .200 | .261 | .052 | .122 | .200 | .241 | .053 | .188 | .200 | .165 | .200 | .173 | .200 | .207 | .131 | .197 | .096 |
| P4 | .180 | .200 | .112 | .200 | .189 | .131 | .144 | .200 | .211 | .146 | .257 | .200 | .133 | .200 | .191 | .200 | .160 | .200 | .204 | .094 |
| P6 | .135 | .200 | .171 | .200 | .180 | .178 | .125 | .200 | .204 | .178 | .207 | .051 | .195 | .190 | .204 | .142 | .233 | .051 | .136 | .200 |
| P8 | .254 | .096 | .201 | .200 | .191 | .082 | .143 | .200 | .166 | .200 | .260 | .036 | .210 | .122 | .227 | .067 | .183 | .200 | .147 | .200 |
| PO7 | .180 | .200 | .217 | .200 | .143 | .200 | .212 | .068 | .253 | .033 | .253 | .047 | .152 | .200 | .156 | .200 | .244 | .034 | .164 | .200 |
| PO5 | .219 | .200 | .228 | .152 | .178 | .138 | .185 | .148 | .194 | .200 | .193 | .200 | .226 | .067 | .167 | .200 | .220 | .086 | .082 | .200 |
| PO3 | .131 | .200 | .151 | .200 | .146 | .200 | .207 | .051 | .158 | .200 | .152 | .200 | .200 | .164 | .201 | .082 | .147 | .200 | .201 | .104 |
| POZ | .217 | .200 | .187 | .200 | .136 | .200 | .198 | .095 | .171 | .200 | .159 | .200 | .181 | .200 | .197 | .096 | .116 | .200 | .158 | .200 |
| PO4 | .172 | .200 | .213 | .111 | .169 | .200 | .191 | .082 | .233 | .072 | .204 | .200 | .212 | .068 | .187 | .137 | .202 | .152 | .237 | .023 |
| PO6 | .278 | .044 | .107 | .200 | .262 | .076 | .143 | .200 | .228 | .085 | .237 | .084 | .206 | .085 | .170 | .200 | .138 | .200 | .173 | .165 |
| PO8 | .225 | .200 | .200 | .086 | .201 | .083 | .178 | .138 | .210 | .148 | .134 | .200 | .238 | .043 | .171 | .200 | .159 | .200 | .150 | .200 |
| O1 | .232 | .176 | .175 | .200 | .133 | .200 | .215 | .046 | .152 | .200 | .191 | .200 | .212 | .113 | .141 | .200 | .226 | .067 | .110 | .200 |
| OZ | .166 | .200 | .212 | .068 | .203 | .076 | .228 | .085 | .131 | .200 | .229 | .112 | .182 | .200 | .193 | .113 | .210 | .119 | .164 | .200 |
| O2 | .240 | .144 | .161 | .200 | .206 | .085 | .176 | .200 | .207 | .164 | .288 | .130 | .197 | .096 | .199 | .089 | .157 | .200 | .154 | .200 |

**Supplementary table8. The mean of θ band**

| Left-hand MI | | | Right-hand MI | | | Feet MI | | | EO | | | EC | | |
| --- | --- | --- | --- | --- | --- | --- | --- | --- | --- | --- | --- | --- | --- | --- |
| Channel | PWP | PWN | Channel | PWP | PWN | Channel | PWP | PWN | Channel | PWP | PWN | Channel | PWP | PWN |
| FP1 | 6.2672 | 8.368325 | FP1 | 6.074135 | 7.567937 | FP1 | 5.830596 | 8.600417 | C1 | 10.18874 | 11.34419 | FP1 | 10.79157 | 12.10846 |
| FPZ | 5.418919 | 7.130841 | FPZ | 5.808595 | 7.267384 | FPZ | 5.643581 | 8.414177 | P8 | 9.953351 | 9.971267 | T7 | 10.64395 | 11.76581 |
| FP2 | 5.835067 | 6.691348 | AF4 | 6.22733 | 7.171719 | AF3 | 6.041558 | 7.709367 |  |  |  |  |  |  |
| AF3 | 5.612695 | 7.569253 | F3 | 5.817165 | 7.386204 | AF4 | 6.23257 | 7.569926 |  |  |  |  |  |  |
| AF4 | 5.242536 | 7.710038 | FZ | 6.461039 | 7.930921 | F5 | 6.343458 | 7.834012 |  |  |  |  |  |  |
| F7 | 5.377956 | 6.652329 | F6 | 6.910307 | 7.882827 | F3 | 5.924144 | 7.801438 |  |  |  |  |  |  |
| F5 | 5.551982 | 7.546504 | F8 | 5.983371 | 7.452152 | F1 | 5.988149 | 7.055625 |  |  |  |  |  |  |
| F3 | 5.133345 | 7.941416 | FC5 | 6.422177 | 7.834159 | FZ | 5.832773 | 8.256113 |  |  |  |  |  |  |
| F1 | 5.702809 | 6.379227 | FC3 | 5.374382 | 7.77828 | F2 | 5.408161 | 8.630721 |  |  |  |  |  |  |
| FZ | 5.914153 | 8.151302 | FCZ | 6.429236 | 7.233011 | F4 | 5.87833 | 7.559396 |  |  |  |  |  |  |
| F2 | 5.533483 | 7.159388 | FC2 | 5.879873 | 7.366015 | F6 | 6.199978 | 8.59811 |  |  |  |  |  |  |
| F6 | 6.097925 | 8.031433 | FT8 | 6.17046 | 7.554881 | F8 | 6.158363 | 8.707231 |  |  |  |  |  |  |
| F8 | 5.402582 | 6.969628 | C5 | 6.377855 | 7.431733 | FT7 | 6.719197 | 7.92354 |  |  |  |  |  |  |
| FT7 | 6.107371 | 6.86601 | C3 | 5.690183 | 6.668107 | FC5 | 6.82611 | 8.866336 |  |  |  |  |  |  |
| FC5 | 6.073197 | 8.067516 | CZ | 5.811954 | 6.869602 | FC3 | 5.917014 | 8.49134 |  |  |  |  |  |  |
| FC3 | 5.641148 | 6.937185 | C2 | 5.518482 | 6.949661 | FC1 | 6.16849 | 7.46392 |  |  |  |  |  |  |
| FCZ | 6.137667 | 7.80403 | C6 | 5.958553 | 6.894698 | FCZ | 5.528636 | 8.160865 |  |  |  |  |  |  |
| FC2 | 5.718747 | 7.338398 | T8 | 5.292708 | 7.813067 | FC2 | 5.544173 | 8.331352 |  |  |  |  |  |  |
| FC4 | 6.039015 | 7.144135 | TP7 | 5.475618 | 6.832888 | FC4 | 5.922487 | 7.291663 |  |  |  |  |  |  |
| FC6 | 5.794385 | 8.271324 | CP5 | 5.790853 | 6.590065 | FC6 | 6.135612 | 7.852167 |  |  |  |  |  |  |
| FT8 | 5.521823 | 7.719795 | CP3 | 5.353099 | 7.188571 | FT8 | 6.220591 | 8.05012 |  |  |  |  |  |  |
| T7 | 5.808048 | 7.218404 | CP1 | 5.483046 | 6.849906 | C5 | 6.341419 | 7.846858 |  |  |  |  |  |  |
| C5 | 6.088938 | 7.998089 | CP2 | 5.565029 | 6.840911 | C3 | 5.677907 | 7.70976 |  |  |  |  |  |  |
| C3 | 5.660626 | 7.550301 | CP4 | 5.687736 | 7.151637 | C1 | 6.124553 | 6.897955 |  |  |  |  |  |  |
| C1 | 6.097305 | 7.254282 | CP6 | 6.476626 | 7.639265 | CZ | 5.85475 | 7.744482 |  |  |  |  |  |  |
| CZ | 6.294825 | 7.992099 | TP8 | 5.66458 | 6.568831 | C2 | 5.690711 | 7.862749 |  |  |  |  |  |  |
| C2 | 5.74623 | 7.849718 | P5 | 6.534706 | 7.562064 | C6 | 6.085229 | 7.641242 |  |  |  |  |  |  |
| C4 | 5.682652 | 7.161532 | P3 | 5.363864 | 6.870366 | T8 | 6.077927 | 8.412617 |  |  |  |  |  |  |
| C6 | 6.640631 | 8.16181 | PZ | 6.070836 | 7.404482 | TP7 | 6.773247 | 7.613224 |  |  |  |  |  |  |
| T8 | 6.069747 | 7.746505 | P4 | 5.930788 | 6.614464 | CP5 | 6.660229 | 7.720804 |  |  |  |  |  |  |
| TP7 | 6.062423 | 7.311791 | P6 | 6.021405 | 7.393867 | CP3 | 6.335949 | 7.95225 |  |  |  |  |  |  |
| CP3 | 5.987371 | 7.06541 | P8 | 5.405015 | 7.022953 | CP2 | 6.139958 | 7.309757 |  |  |  |  |  |  |
| CP2 | 5.168244 | 6.532582 | PO7 | 5.667319 | 7.209544 | CP6 | 6.60733 | 8.171414 |  |  |  |  |  |  |
| CP6 | 6.056416 | 7.695774 | PO5 | 5.96426 | 7.01417 | TP8 | 6.286988 | 7.771279 |  |  |  |  |  |  |
| TP8 | 5.479292 | 7.050498 | PO3 | 5.127307 | 6.025563 | P5 | 6.362545 | 8.374654 |  |  |  |  |  |  |
| P5 | 6.105065 | 8.656075 | POZ | 5.400376 | 6.793375 | P3 | 6.018767 | 7.493404 |  |  |  |  |  |  |
| P3 | 5.534938 | 7.389018 | PO4 | 5.756753 | 7.270635 | PZ | 5.977984 | 8.539336 |  |  |  |  |  |  |
| PZ | 6.591563 | 8.021504 | PO6 | 5.155581 | 6.097482 | P4 | 6.178334 | 7.091803 |  |  |  |  |  |  |
| P6 | 6.162185 | 7.581845 | PO8 | 5.592748 | 6.875793 | P6 | 6.35245 | 7.146117 |  |  |  |  |  |  |
| P8 | 5.647979 | 7.727155 | O1 | 6.222339 | 7.742446 | P8 | 6.17267 | 7.818159 |  |  |  |  |  |  |
| PO7 | 5.69253 | 7.485318 | OZ | 5.612341 | 6.562499 | PO7 | 6.367366 | 7.309592 |  |  |  |  |  |  |
| PO5 | 5.079512 | 7.804359 |  |  |  | PO5 | 6.542281 | 7.574986 |  |  |  |  |  |  |
| PO3 | 4.85678 | 6.728974 |  |  |  | PO3 | 5.956973 | 7.152727 |  |  |  |  |  |  |
| POZ | 5.168004 | 7.083608 |  |  |  | POZ | 6.010997 | 6.799173 |  |  |  |  |  |  |
| PO4 | 5.365399 | 8.227557 |  |  |  | PO4 | 6.192414 | 8.269712 |  |  |  |  |  |  |
| PO6 | 5.268165 | 6.440352 |  |  |  | PO6 | 5.91274 | 7.331001 |  |  |  |  |  |  |
| PO8 | 5.58216 | 7.744907 |  |  |  | PO8 | 6.291064 | 7.58795 |  |  |  |  |  |  |
| O1 | 6.138386 | 8.298346 |  |  |  | O1 | 6.109642 | 8.474078 |  |  |  |  |  |  |
| OZ | 5.494395 | 6.831375 |  |  |  | OZ | 5.660952 | 7.774231 |  |  |  |  |  |  |
| O2 | 5.713949 | 6.98834 |  |  |  | O2 | 6.028383 | 7.296785 |  |  |  |  |  |  |

**Supplementary table9. The mean of α band**

| Left-hand MI | | | Right-hand MI | | | Feet MI | | | EO | | | EC | | |
| --- | --- | --- | --- | --- | --- | --- | --- | --- | --- | --- | --- | --- | --- | --- |
| Channel | PWP | PWN | Channel | PWP | PWN | Channel | PWP | PWN | Channel | PWP | PWN | Channel | PWP | PWN |
| FP1 | 5.985293 | 7.517216 | FP1 | 6.250557 | 6.944162 | FP1 | 6.692913 | 7.702161 | F8 | 4.848955 | 8.670704 | FPZ | 6.005816 | 8.64591 |
| FPZ | 5.770355 | 7.364715 | FPZ | 5.684453 | 7.572378 | FPZ | 5.575977 | 7.314206 | FC1 | 4.681804 | 8.92457 | FP2 | 5.219031 | 8.042468 |
| FP2 | 5.780088 | 6.405258 | F3 | 5.666048 | 7.25403 | FP2 | 5.472906 | 6.09157 | FCZ | 4.772586 | 8.655909 | AF3 | 4.873626 | 8.925 |
| AF3 | 6.311269 | 7.5581 | FZ | 6.546433 | 7.857134 | AF3 | 6.386625 | 7.178435 | FC4 | 4.087128 | 6.874675 | F5 | 4.710336 | 8.420558 |
| AF4 | 5.647459 | 6.756206 | F2 | 5.723009 | 6.982581 | AF4 | 5.620061 | 6.669948 | FC6 | 4.984514 | 8.582989 | F3 | 5.172188 | 9.76259 |
| F7 | 5.509843 | 6.305065 | F4 | 5.526892 | 6.277918 | F5 | 6.626532 | 7.489653 | FT8 | 4.608489 | 9.746646 | F1 | 4.635524 | 8.755554 |
| F5 | 6.136073 | 7.427152 | F8 | 5.563976 | 7.271393 | F3 | 5.47035 | 6.754509 | T7 | 4.229573 | 9.170221 | FZ | 4.862217 | 8.065343 |
| F3 | 6.07739 | 6.861666 | FT7 | 5.476338 | 6.289168 | F1 | 5.314365 | 6.219968 | C5 | 6.080873 | 8.943227 | F2 | 5.752597 | 8.83258 |
| F2 | 6.397526 | 7.500424 | FC5 | 6.822238 | 8.36587 | F2 | 5.581646 | 7.210523 | C3 | 4.912281 | 10.03312 | F6 | 5.326445 | 8.729168 |
| F6 | 6.582534 | 7.304098 | FC3 | 5.582164 | 6.542566 | F4 | 5.069815 | 6.164532 | C1 | 4.940518 | 10.01321 | F8 | 5.833846 | 8.987829 |
| F8 | 5.967343 | 7.534373 | FC2 | 5.674013 | 7.19018 | F6 | 6.591668 | 7.430327 | CZ | 6.756545 | 9.263675 | FC5 | 4.554075 | 7.911064 |
| FC5 | 6.386992 | 8.442887 | FT8 | 5.885065 | 8.365532 | F8 | 5.818521 | 7.795962 | C2 | 4.828791 | 9.463658 | FC3 | 4.936352 | 8.636759 |
| FC3 | 5.618784 | 7.335994 | T7 | 5.82589 | 6.553698 | FT7 | 5.719655 | 6.448556 | C4 | 5.876509 | 9.705483 | FC1 | 6.274515 | 9.850807 |
| FC1 | 5.785704 | 6.999736 | C5 | 6.320518 | 7.991685 | FC5 | 6.81534 | 8.060314 | P3 | 5.488486 | 9.616403 | FCZ | 6.114839 | 9.421008 |
| FC2 | 6.305886 | 7.56439 | C1 | 5.162209 | 5.852637 | FC3 | 6.102138 | 6.726721 | PZ | 5.162748 | 6.898333 | FC4 | 4.2943 | 7.513621 |
| FC6 | 6.178351 | 7.9308 | C2 | 5.259312 | 6.921585 | FC1 | 5.449697 | 6.364347 | P4 | 5.326697 | 10.01809 | FC6 | 5.469711 | 9.670645 |
| FT8 | 5.320581 | 7.809963 | C4 | 5.338668 | 6.202408 | FC2 | 5.789352 | 7.423815 | PO7 | 4.602524 | 7.589858 | FT8 | 4.513039 | 9.866631 |
| T7 | 5.001812 | 6.64475 | C6 | 5.773411 | 6.520917 | FT8 | 6.197555 | 7.387826 | PO5 | 5.372042 | 9.972279 | C3 | 4.659608 | 12.27352 |
| C5 | 6.450613 | 7.874918 | T8 | 5.524357 | 7.394651 | T8 | 6.176997 | 7.248202 | PO3 | 4.765146 | 11.04599 | C1 | 4.578357 | 9.98833 |
| C2 | 5.62879 | 7.182451 | TP7 | 4.994931 | 6.076367 | CP5 | 7.627329 | 5.981739 | POZ | 4.653998 | 9.816598 | C2 | 4.492289 | 8.482971 |
| C4 | 5.099863 | 6.849165 | CP3 | 5.26093 | 6.664739 | CP1 | 6.6687 | 5.486853 | PO6 | 5.739573 | 11.33149 | C4 | 3.949154 | 9.696257 |
| C6 | 6.455672 | 7.123894 | CP2 | 5.481318 | 6.470806 | TP8 | 7.035579 | 6.127332 | PO8 | 5.265881 | 11.09639 | TP7 | 5.355728 | 8.455132 |
| T8 | 5.770058 | 7.567161 | CP6 | 6.04721 | 7.583475 | P7 | 6.382825 | 5.720947 | OZ | 5.573356 | 10.51979 | CP5 | 5.735332 | 9.528058 |
| TP7 | 5.52053 | 6.446858 | TP8 | 5.429464 | 6.413991 | P6 | 7.307815 | 7.307815 | O2 | 6.880128 | 10.55385 | CP2 | 5.298327 | 9.892494 |
| CP3 | 5.638922 | 6.612974 | P7 | 4.775822 | 5.788253 | POZ | 5.372009 | 5.372009 |  |  |  | CP4 | 5.099882 | 9.329606 |
| CP2 | 5.50902 | 6.278522 | P5 | 5.727309 | 6.808411 |  |  |  |  |  |  | P5 | 5.773547 | 8.992927 |
| CP4 | 5.287942 | 6.160891 | P3 | 5.069832 | 5.724243 |  |  |  |  |  |  | PZ | 4.969362 | 8.196057 |
| CP6 | 6.108701 | 7.400241 | PZ | 5.984861 | 7.398395 |  |  |  |  |  |  | P4 | 6.40015 | 10.42045 |
| TP8 | 5.676174 | 6.511002 | P4 | 5.059943 | 5.988991 |  |  |  |  |  |  | PO5 | 6.318818 | 10.27761 |
| P3 | 5.78772 | 7.180692 | P6 | 6.062807 | 6.953533 |  |  |  |  |  |  | PO3 | 5.136741 | 10.36401 |
| PZ | 6.336375 | 7.429614 | P8 | 5.341639 | 6.557155 |  |  |  |  |  |  | PO6 | 5.02441 | 12.67727 |
| P4 | 5.663461 | 6.347762 | PO7 | 5.205053 | 6.624698 |  |  |  |  |  |  | PO8 | 5.187768 | 10.49681 |
| P6 | 6.303723 | 7.358003 | POZ | 5.498847 | 6.170013 |  |  |  |  |  |  | OZ | 4.985983 | 8.494651 |
| P8 | 5.767929 | 7.474366 | PO4 | 5.707624 | 7.174258 |  |  |  |  |  |  | O2 | 4.491178 | 9.597929 |
| PO7 | 5.55396 | 7.264047 | PO8 | 5.350874 | 5.914307 |  |  |  |  |  |  |  |  |  |
| PO5 | 5.927452 | 7.035187 | O1 | 7.147434 | 6.21086 |  |  |  |  |  |  |  |  |  |
| PO3 | 5.344939 | 6.676051 | OZ | 6.114247 | 5.53137 |  |  |  |  |  |  |  |  |  |
| POZ | 5.08386 | 6.500242 |  |  |  |  |  |  |  |  |  |  |  |  |
| PO4 | 5.771757 | 6.995011 |  |  |  |  |  |  |  |  |  |  |  |  |
| PO6 | 5.642766 | 6.233111 |  |  |  |  |  |  |  |  |  |  |  |  |
| PO8 | 5.663568 | 6.850947 |  |  |  |  |  |  |  |  |  |  |  |  |
| OZ | 5.776409 | 6.565427 |  |  |  |  |  |  |  |  |  |  |  |  |
| O2 | 5.282682 | 6.238981 |  |  |  |  |  |  |  |  |  |  |  |  |

**Supplementary table10. The mean of β band**

| Left-hand MI | | | Right-hand MI | | | Feet MI | | | EO | | | EC | | |
| --- | --- | --- | --- | --- | --- | --- | --- | --- | --- | --- | --- | --- | --- | --- |
| Channel | PWP | PWN | Channel | PWP | PWN | Channel | PWP | PWN | Channel | PWP | PWN | Channel | PWP | PWN |
| FPZ | 5.912704 | 4.811945 | FP1 | 4.854229 | 4.298991 | FP1 | 5.855784 | 4.564288 | FP2 | 9.269405 | 4.3374 | FP1 | 8.63928 | 4.606785 |
| FP2 | 5.615722 | 4.846134 | AF3 | 5.522771 | 4.587694 | FPZ | 5.679061 | 5.085094 | AF3 | 8.807876 | 4.273195 | FPZ | 8.719994 | 5.653579 |
| AF3 | 5.492309 | 4.646125 | AF4 | 5.72687 | 4.859128 | FP2 | 6.0363 | 4.93832 | F7 | 10.119 | 5.56637 | FP2 | 9.269405 | 4.3374 |
| AF4 | 5.699384 | 4.931461 | F7 | 5.793576 | 5.13788 | AF3 | 5.591268 | 4.047175 | F4 | 9.305571 | 4.860053 | AF3 | 8.807876 | 4.273195 |
| F5 | 6.159982 | 5.047524 | F5 | 5.891769 | 4.343135 | AF4 | 5.90358 | 4.926019 | F6 | 9.815481 | 5.273398 | F7 | 10.119 | 5.56637 |
| F3 | 6.454887 | 4.751781 | FZ | 5.424884 | 4.737465 | F5 | 5.743342 | 4.905196 | FC5 | 9.440249 | 3.830745 | F3 | 8.877567 | 4.938718 |
| F1 | 5.528397 | 4.768958 | F2 | 5.901517 | 5.108821 | FZ | 6.063245 | 4.734519 | FC3 | 9.866151 | 4.916925 | F1 | 7.997924 | 5.879148 |
| FZ | 6.00935 | 4.686734 | F4 | 5.583346 | 4.819267 | F2 | 5.344081 | 4.524558 | CP5 | 11.41921 | 6.156842 | FZ | 10.1251 | 6.035284 |
| F2 | 6.136657 | 5.263336 | F6 | 5.702843 | 4.665398 | F6 | 6.424103 | 4.785587 |  |  |  | F2 | 9.418588 | 4.922366 |
| F4 | 5.846335 | 5.019248 | FT7 | 5.773755 | 4.949704 | F8 | 5.553468 | 4.901188 |  |  |  | F4 | 9.305571 | 4.860053 |
| F6 | 5.862485 | 4.607358 | FC5 | 6.215947 | 5.115789 | FC5 | 5.666876 | 4.829778 |  |  |  | F6 | 9.815481 | 5.273398 |
| F8 | 6.087944 | 5.088229 | FC3 | 5.580209 | 4.991098 | FC1 | 5.788698 | 5.029202 |  |  |  | F8 | 10.54703 | 6.061156 |
| FC5 | 6.231799 | 5.077654 | FCZ | 5.65678 | 4.822229 | FCZ | 5.674895 | 4.913597 |  |  |  | FT7 | 9.632951 | 6.766263 |
| FC3 | 5.708658 | 5.0459 | FC2 | 5.957503 | 4.969654 | C5 | 5.632404 | 4.339523 |  |  |  | FC5 | 9.440249 | 3.830745 |
| FC1 | 5.367991 | 4.572637 | FC4 | 5.62567 | 4.76791 | CZ | 5.933667 | 4.782881 |  |  |  | FC3 | 9.866151 | 4.916925 |
| FCZ | 6.06259 | 4.925939 | FC6 | 5.824602 | 5.006822 | C2 | 5.674746 | 4.979829 |  |  |  | FC1 | 9.62676 | 5.348128 |
| FC2 | 5.912803 | 4.604646 | FT8 | 6.062805 | 5.383528 | C4 | 6.328705 | 5.052012 |  |  |  | FCZ | 9.670225 | 5.959514 |
| FC4 | 5.287239 | 4.747585 | C5 | 5.956117 | 4.912058 | C6 | 6.367009 | 4.480758 |  |  |  | FC2 | 9.316323 | 4.964205 |
| FC6 | 5.86305 | 4.868356 | C3 | 6.346544 | 5.041633 | T8 | 5.885726 | 4.915396 |  |  |  | FC4 | 7.654798 | 6.386982 |
| FT8 | 5.744159 | 5.090969 | CZ | 5.262559 | 4.498606 | CP5 | 5.682127 | 4.379474 |  |  |  | FT8 | 10.05329 | 5.949449 |
| C5 | 6.311159 | 4.848804 | C2 | 5.828089 | 4.988324 | CP3 | 6.254444 | 5.145931 |  |  |  | T7 | 11.00289 | 6.240228 |
| C3 | 5.883897 | 5.213295 | C6 | 6.134668 | 4.865801 | CP1 | 6.06408 | 4.860837 |  |  |  | C5 | 8.775882 | 6.127042 |
| C1 | 5.466427 | 4.662954 | CP1 | 5.767516 | 4.570981 | CP2 | 5.831737 | 4.868423 |  |  |  | C3 | 11.20392 | 7.057864 |
| C2 | 5.582379 | 5.016023 | CP6 | 5.561746 | 4.701292 | CP4 | 5.753531 | 4.721008 |  |  |  | C1 | 10.08858 | 5.585236 |
| C6 | 5.953558 | 4.794749 | P5 | 5.3415 | 4.610055 | CP6 | 5.945147 | 4.74312 |  |  |  | C2 | 7.926981 | 7.291227 |
| T8 | 6.098061 | 4.672356 | PZ | 5.685792 | 4.567194 | TP8 | 5.746052 | 4.889575 |  |  |  | C4 | 9.664196 | 6.219062 |
| CP3 | 5.37309 | 4.220893 | P6 | 6.28844 | 4.302304 | P7 | 5.735021 | 4.701554 |  |  |  | TP7 | 10.22256 | 6.622614 |
| CP6 | 5.92941 | 4.744526 | P8 | 5.95069 | 5.118961 | P5 | 6.034341 | 4.496696 |  |  |  | CP1 | 11.12668 | 6.824488 |
| TP8 | 5.654908 | 4.863197 | PO5 | 5.732807 | 4.859074 | PZ | 5.706073 | 4.738428 |  |  |  | CP4 | 9.339049 | 6.130398 |
| P7 | 5.885432 | 4.694148 | PO3 | 6.141758 | 5.108719 | P4 | 5.547391 | 4.329712 |  |  |  | TP8 | 10.22426 | 5.913138 |
| P5 | 5.386735 | 4.687518 | PO6 | 5.608034 | 4.703884 | P8 | 5.770151 | 5.207182 |  |  |  | P5 | 9.573869 | 6.520983 |
| P3 | 5.73509 | 5.172529 | PO8 | 5.219232 | 5.1676 | PO7 | 5.595342 | 4.440847 |  |  |  | PO3 | 9.537635 | 6.983033 |
| P4 | 5.701548 | 4.862114 | O1 | 5.841352 | 4.097891 | PO5 | 5.719647 | 4.916646 |  |  |  | POZ | 11.11897 | 6.65491 |
| PO7 | 5.331997 | 4.689853 | OZ | 5.616631 | 4.80785 | PO3 | 5.687759 | 4.922951 |  |  |  | PO6 | 12.65715 | 8.483619 |
| PO5 | 5.656015 | 5.038759 |  |  |  | PO4 | 5.765113 | 4.864242 |  |  |  | PO8 | 9.919558 | 6.312114 |
| PO4 | 5.529942 | 4.854331 |  |  |  | PO6 | 5.815019 | 5.055169 |  |  |  |  |  |  |
| PO6 | 5.65327 | 4.822083 |  |  |  | PO8 | 5.760081 | 5.037559 |  |  |  |  |  |  |
| O1 | 5.990702 | 4.982895 |  |  |  | O1 | 5.584627 | 4.528105 |  |  |  |  |  |  |
| OZ | 5.650249 | 4.899977 |  |  |  | OZ | 5.398521 | 4.72654 |  |  |  |  |  |  |

**Supplementary table13. Z and T value of θ band during feet MI**

**Supplementary table14. Z and T value of α band in the resting state with EO**

**Supplementary table15. Z and T value of β band in the resting state with EO**

**Supplementary table16. Z and T value of θ band in the resting state with EO**

# Supplementary formula

The formula of CWT is:

$W\left( \mu，d \right)=[x\left( t \right),\varphi_{\mu,d}\left( t \right)]=\frac{1}{\sqrt{d}}\int_{-\infty}^{+\infty} g(t)\varphi\bullet(\frac{t-\mu}{d})dt$ (1)

where $W\left( \mu，d \right)$ is the wavelet transform coefficient. $(\frac{t-\mu}{d})dt$ is called the wavelet basis function. The wavelet base has two parameters: translation factor μ and scale factor d, d is called the wavelet scale and is a parameter representing the frequency of the EEG signal, and μ is a parameter representing the time of the EEG signal.

 Where the scale factor $d=\frac{1}{f}$.

The formula of MST is:

$S(\mu，n)=\int_{-\infty}^{+\infty} g(t)\gamma(\mu-t，n)exp(-j2\pi nt)dt$ (2)

where g(t) is the EEG signal, $n$ is the frequency of signal, μ is the parameter controlling the location of the Gaussian window function on the time axis t, and $\gamma(\mu-t，n)$ is the Gaussian window function. The formula is as follows:

$\gamma\left( \tau-t,n \right)=\frac{1}{\sqrt{2\pi}\sigma_{2}\left( f \right)}exp(\frac{{-(\tau-t)}^{2}}{2\sigma_{2}^{2}\left( f \right)})$ (3)

where $\sigma_{2}\left( f \right)$ is the standard deviation and it is defined as follows:

$\sigma_{2}\left( f \right)=\frac{p}{\left| n \right|^{q}}$ (4)

where p and q are adaptive parameters.

The normalization formula is as follows:

$x=\frac{x-x_{\min}}{x_{\max}-x_{\min}}$ (5)

where $x_{\min}$ is the minimum of PSD, $x_{\max}$ is the maximum of PSD.

#### Brain network

The PLV formula is:

PLV=$⎸\frac{1}{N}\sum_{j=0}^{n-1} e^{i\Delta\lambda(t)}⎹$ ,Δλ(t)=$\lambda_{g}\left( j\Delta t \right)-\lambda_{p}(j\Delta t)$ (6)

where t is the point of time, and n is the number of samples per signal. Δλ(t) represents the phase difference of signal g(t) and p(t). Δt represents the sampling period. j represents the jth sampling point. The network has been constructed with 21 electrodes (Fp1, Fpz, Fp2, F7, F3, Fz, F4, F8, T7, C3, Cz, C4, T8, P7, P3, Pz, P4, P8, O1, Oz, O2) as the network nodes.
